# The impact of e-cigarette use on periodontal health: a systematic review and meta-analysis

**DOI:** 10.1038/s41432-025-01119-6

**Published:** 2025-02-13

**Authors:** Rajpal Tattar, Joshua Jackson, Richard Holliday

**Affiliations:** 1 The School of Clinical Dentistry, University of Sheffield, Sheffield, UK; 2 School of Dental Sciences, Faculty of Medical Sciences, Newcastle University, Newcastle Upon Tyne, UK; 3 Newcastle Upon Tyne Hospitals NHS Foundation Trust, Newcastle Upon Tyne, UK

**Keywords:** Periodontics, Periodontitis

## Abstract

**Background:**

Electronic Nicotine Delivery Systems (ENDS, e-cigarettes) are a popular alternative to traditional tobacco smoking. Objective: To evaluate the effect of ENDS use on periodontal health.

**Methods:**

A protocol was published in accordance with PRISMA standards. Subjects with periodontal health, gingivitis and periodontitis were included. Reviews, case reports, letters, abstracts, narratives and expert opinions were excluded. Databases searched included PubMed, Embase, Web of Science, CINAHL Plus, and Dentistry & Oral Sciences Source up until February 2024. Risk of bias was evaluated using the Newcastle-Ottawa Scale, ROBINS-I and the RoB 2 tools.

**Results:**

40 eligible studies were included. Smokers had poorer clinical outcomes than ENDS users and non-smokers/former smokers, apart from bleeding on probing and gingival indices. There was no difference between ENDS users and non-smokers/former smokers in markers of periodontal destruction (pocket probing depths/marginal bone loss). ENDS users had higher plaque scores than non-smokers/former smokers. ENDS use leads to unique microbial changes compared to tobacco smokers and higher pro-inflammatory markers compared to non-smokers/former smokers.

**Conclusion:**

Within the limitations of the included studies which are at high risk of bias, we found evidence that ENDS use had some impact on periodontal parameters compared to non-smokers/former smokers. Tobacco smokers had consistently worst outcomes.

**Registration PROSPERO 2024:**

CRD42024496560.

Key points

- Studies conducted within this field are at high risk of bias (usually due to cofounding factors) and so caution must be applied to the conclusions generated
- There is evidence to suggest that ENDS use had some impacts on periodontal parameters compared to non-smokers/former smokers. Tobacco smokers had consistently worst outcomes.
- There was no evidence of a difference between ENDS users and non-smokers/former smokers in markers of periodontal destruction (pocket probing depths/marginal bone loss)
- Further well-designed research is required in this field to inform clinical practice and guidelines

## Introduction

In recent years, electronic cigarettes (referred to as ENDS [Electronic Nicotine Delivery Systems] hereon) have become a popular alternative to traditional tobacco smoking. They are reported as less harmful due to the absence of combustion and many toxicants.

Periodontal health is fundamental to both oral and systemic well-being, with disturbances potentially leading to conditions such as gingivitis, periodontitis, and eventual tooth loss. Traditional tobacco smoking is well known to harm periodontal tissues^1^. However, the effects of ENDS are still being studied. ENDS expose periodontal tissues to aerosolized nicotine and chemicals, which may affect oral health differently than tobacco smoking. Given the growing popularity of ENDS, especially among younger people, further research is needed to understand any effects on oral health and to guide public health initiatives and clinical guidelines.

There have been several previous narrative reviews, systematic reviews and meta-analysis investigating the impact of ENDS use on periodontal health^2–8^. Their conclusions have ranged from ENDS causing ‘increased destruction of the periodontium leading to the development of disease’ to periodontal parameters being similar among non-smokers and END users. Most systematic reviews have conducted a risk of bias or quality assessment of the included studies (Table S4, supplementary materials). Although well-known tools are often used for this assessment the main methodological issue in this field (confounding from tobacco smoking) has not previously been addressed apart from in discussion. ENDS users are often current or former tobacco smokers making it hard to differentiate the impact of ENDS use alone on the outcomes of interest and resulting in  misleading conclusions. In this review we plan to assess the included studies on this issue.

This review takes a comprehensive approach to assessing the effects of ENDS use on periodontal health, unlike previous reviews that focused on single outcomes. It evaluates patient-reported outcomes, clinical parameters, oral microbiome changes, and immunological responses. By synthesising current evidence, this review aims to elucidate any evidence on the relationship between ENDS use and periodontal health.

## Materials and methods

This systematic review, registered with PROSPERO (ID CRD42024496560) on 1st March 2024, adheres to the Preferred Reporting Items for Systematic Review and Meta-Analyses (PRISMA) guidelines^9^. It examines the impact of ENDS on individuals with healthy periodontium, gingivitis, or periodontitis. The primary outcomes analysed were changes in periodontal parameters (Pocket Probing Depth, PPD; Marginal Bone Loss, MBL; Clinical Attachment Level, CAL; Gingival Indices, GI; Bleeding on Probing, BOP; and plaque indices, PI), while secondary outcomes included Patient Reported Outcome Measures (PROMS), microbiological changes, and levels of biological markers. The review included observational and interventional studies restricted to human subjects and English-language publications.

A search strategy was piloted to ensure high sensitivity over high precision, refined, and used to search the PubMed/MEDLINE, Embase, Web of Science, CINAHL Plus, and Dentistry & Oral Sciences Source databases (see supplementary materials for search strategies). Electronic databases were searched up to February 29, 2024, with no year restrictions, using controlled vocabulary (MeSH) and free text terms. Study selection and data extraction were conducted independently by two reviewers (RT and JJ) using Covidence systematic review software (Veritas Health Innovation, Melbourne, Australia) and standardised Excel data extraction forms, with any discrepancies resolved through discussion with an additional reviewer (RH).

Risk of bias (RoB) was evaluated using the Cochrane ‘Risk of Bias in Non-randomised Studies - of Interventions’ (ROBINS-I) tool for interventional studies and the RoB 2 tool for randomized controlled trials. For observational studies, the ‘Newcastle-Ottawa Scale’ (NOS) was used and adapted, following previously published methodologies to better suit cross-sectional studies and studies investigating the effects of ENDS use on periodontal health (see supplementary materials for adapted NOS RoB Tools used)^10^. RoB assessments were completed by one author (RT) with any areas of uncertainty reviewed by a second (RH) and third (JJ) author as required. Data was synthesized using RevMan Web (Version 8.4.1, 27.08.24), employing a random-effects model for pooling studies. Subgroup analysis was conducted to assess the impact of confounding risk from tobacco smoking in ENDS studies, categorizing them based on biochemical verification or self-reporting with varying levels of confounding risk. Heterogeneity was assessed using the I² statistic. Funnel plots were used to assess reporting bias in meta-analysed outcome measures that included at least 10 studies. Full details of the materials and methods used in this review are available in the supplementary materials.

## Results

**Fig. 1:**
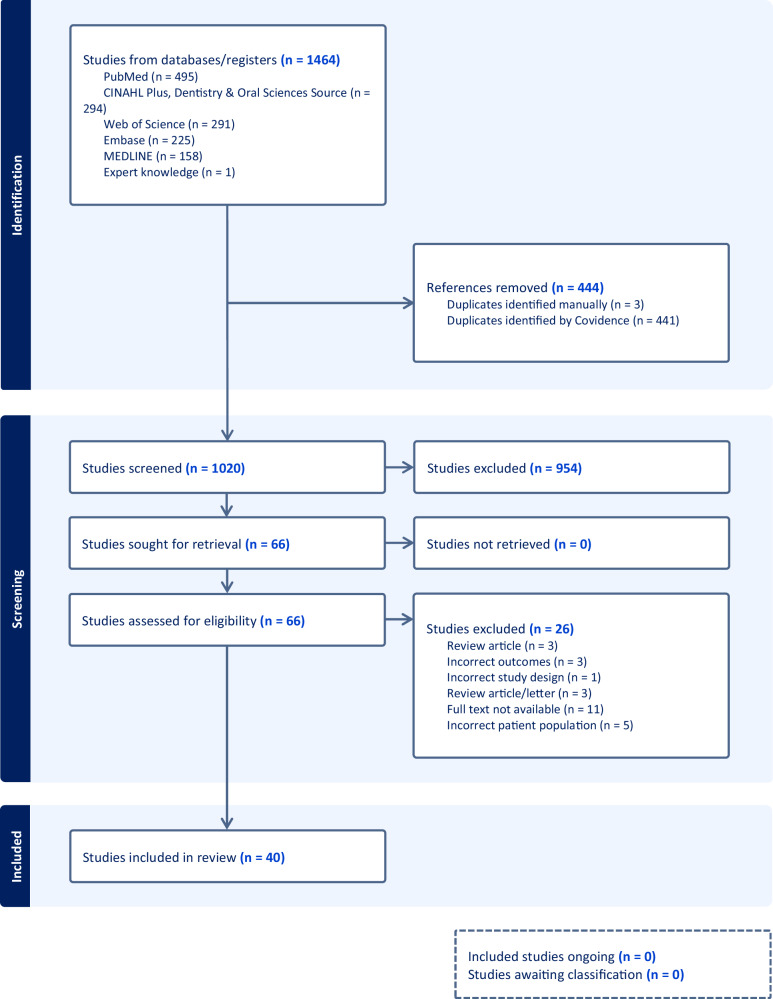
Study flow diagram.

The search strategy yielded 1464 citations. After removing duplicates, 1020 citations were screened, resulting in the identification of 40 eligible articles with 18 included in the meta-analyses (Fig. 1).

### Risk of bias

Many of the included studies were categorised as unsatisfactory or high risk of bias (57.5%, *n* = 23) with 40% deemed satisfactory (*n* = 16) and one study categorised as low risk of bias. Figure 5 provides a summary of the risk of bias assessments (see Tables S1–S3 in supplementary materials for full details).

### Clinical outcomes

**Fig. 2:**
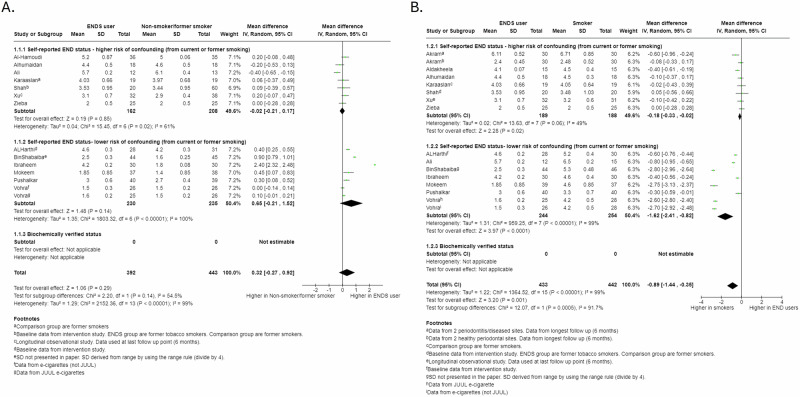
Forest plot of Pocket Probing Depth (PPD) comparisons for cross-sectional data: sub-grouped by risk of tobacco smoking confounding in ENDS group. **A** ENDS users verses non-smokers/former smokers. **B** ENDS verses smokers.

We pooled 15 studies evaluating PPDs in ENDS users. We found no evidence for a difference in PPDs between ENDS users and non-smokers/former smokers (MD 0.32, 95% CI −0.27 to 0.92), although there was a slight trend towards higher PPDs in the ENDS group (Fig. 2A). We found evidence that PPDs were higher in smokers compared to ENDS users (MD −0.89, 95% CI −1.44 to −0.35), with studies at lower risk of confounding reporting greater difference (*P* = 0.0005; Fig. 2B). Following periodontal interventions, we found evidence that ENDS users had higher PPDs than non-smokers/former smokers (MD 1.19, 95% CI 0.26 to 2.12), though this finding was based on only three studies and had a wide confidence interval (Figure S1, supplementary materials). No evidence of a difference was found between smokers and ENDS users in response to periodontal interventions (MD −0.32, 95%CI −1.41 to 0.78), although higher PPDs were suggested in smokers in the study with the lowest risk of confounding (Fig. S2, supplementary materials).

We pooled 13 studies evaluating CAL in ENDS users. We found evidence that CAL was higher in END users compared to non-smokers/former smokers (SMD 1.43, 95% CI 0.48 to 2.38, (Fig. S5, supplementary materials) and higher in smokers compared to ENDS users (SMD −1.24, 95% CI −2.00 to −0.49; Fig. S6, supplementary materials).

We pooled 9 studies evaluating mesial or distal marginal bone loss (MBL). We found no evidence of a difference between ENDS users and non-smokers/former smokers for distal MBL (SMD 0.80, 95% CI −0.50 to 2.11; Fig. S7, supplementary materials) and borderline evidence for a difference for mesial MBL (SMD 1.10, 95%CI 0.01 to 2.19; Figure S8, supplementary materials). We found evidence that smokers had higher mesial and distal MBL than ENDS users (SMD −1.40, 95% CI −2.40 to −0.40; Figure S9, supplementary materials; SMD −1.42, 95% CI −2.49 to −0.36; Fig. S10, supplementary materials).

**Fig. 3:**
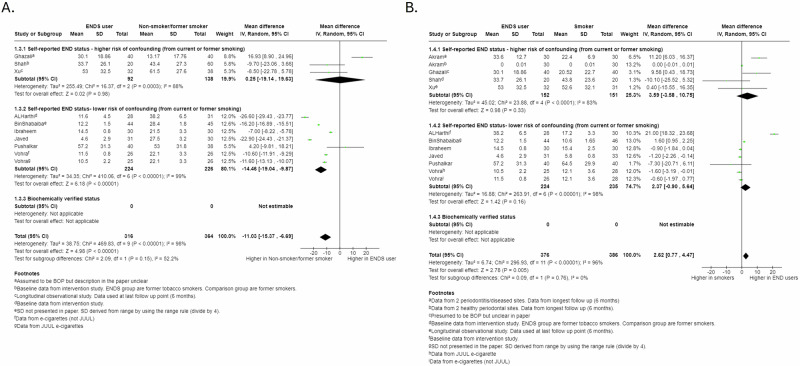
Forest plot of Bleeding on Probing (BOP) comparisons for cross-sectional data: sub-grouped by risk of tobacco smoking confounding in ENDS group. **A** ENDS users verses non-smokers/former smokers. **B** ENDS verses smokers.

We pooled 10 studies evaluating BOP and 8 evaluating gingival indices. We found evidence that non-smokers/former smokers had higher BOP and GI than ENDS users (BOP: MD −11.03, 95%CI −15.37 to −6.69, Fig. 3A; GI: SMD −6.56, 95%CI −9.26 to −3.86; Fig. S11, supplementary materials) with studies at lower risk of confounding suggesting greater difference (for BOP only, *p* = 0.15). Similar effects were seen between smokers and ENDs users (BOP: MD 2.62, 95%CI 0.77 to 4.47, Fig. 3B; GI: SMD 1.22, 95%CI 0.09 to 2.36; Figure S12, supplementary materials). Post-intervention data on BOP and GI was available from only two studies for each (Figs. S13–S16, supplementary materials). Conflictingly, a pilot experimental gingivitis study (not included in the meta-analysis) reported ENDS users had a similar response to non-smokers, suggesting ENDS use did not have the same suppressive effects as tobacco smoke^11^.

**Fig. 4:**
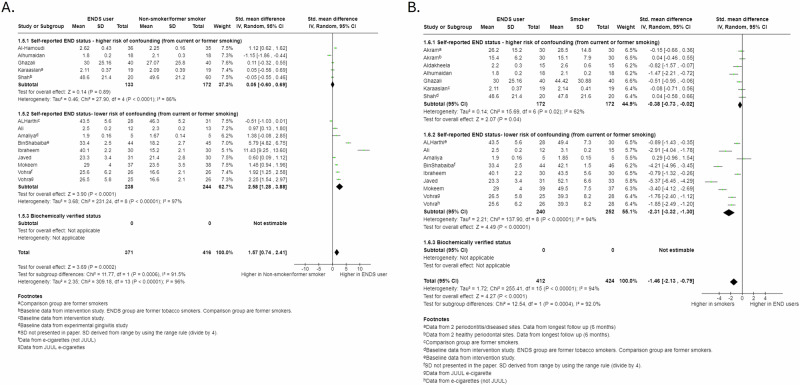
Forest plot of Plaque Indices (PI) comparisons for cross-sectional data: sub-grouped by risk of tobacco smoking confounding in ENDS group. **A** ENDS users verses non-smokers/former smokers. **B** ENDS verses smokers.

We pooled 15 studies evaluating plaque levels. We found evidence that plaque levels were higher in ENDS users compared to non-smokers/former smokers (SMD 1.57, 95%CI 0.74 to 2.41, Fig. 4A) with the effect being more pronounced in studies with lower risk of confounding (*p* = 0.0006). We found evidence that smokers had higher plaque levels than ENDS users (SMD −1.46, 95% CI −2.13 to −0.79, Fig. 4B), with the effect being more pronounced in groups with a lower risk of confounding (*p* = 0.0004). Post-intervention data continued to demonstrate higher plaque levels in ENDS users compared to non-users (SMD 6.25, 95% CI 2.91 to 9.58; Fig. S17, supplementary materials), while no significant difference in plaque levels was found between smokers and ENDS users following periodontal treatment (SMD −1.49, 95%CI −3.52 to 0.54; Fig.S18, supplementary materials).

Two further clinical studies were not included in our meta-analyses. A pilot randomised controlled trial using ENDS as a quit aid in patients with periodontitis reported observing similar improvements in oral health outcomes in those who were provided an ENDS compared to those who were not^12^. A definitive trial is ongoing^13^. A separate study followed smokers switching to ENDS who reported a progressive improvement in periodontal indices^14^.

### Patient-reported outcome measures (PROMs)

Seven studies investigated the association between PROMS and ENDS use, with four of these utilizing data from the Population Assessment of Tobacco and Health (PATH) study^15^. The PATH study is a national longitudinal study examining the impact of tobacco use on health in the United States. Collectively, the four studies utilising this data found that ENDS users have elevated risks of gingival disease and other oral health issues, with significantly higher odds of poor periodontal health, including bone loss^16–19^.

Other research also points to oral health risks associated with smoking and ENDS use. Jeong et al.^20^, using data from the Korean National Health and Nutrition Examination Survey, found that both vapers and smokers had a higher prevalence of periodontal disease than non-users, particularly among men^20^. Similarly, Wiernik et al.^21^ showed, utilising the CONSTANCES cohort, that e-cigarette use increased the risk of severe periodontitis^22^. AlQobaly et al.^22^ analysed NHANES data and found that ENDS users reported higher odds of periodontal disease than non-users, although further analysis showed this could be explained by smoking status^22^. Therefore, caution is warranted in interpreting the findings from the included PROMs studies, as ENDS use was assessed solely through self-reported methods without biochemical verification.

### Biological outcomes

The five studies examining biological markers included in this review collectively highlight the inflammatory impact of smoking and ENDS use on oral health. Ye et al. (2020) found that cigarette smokers had significantly elevated levels of prostaglandin E2 compared to ENDS users and dual users, though there were no significant differences between ENDS users, dual users, and non-smokers^23^. Additionally, markers like myeloperoxidase (MPO) and matrix metalloproteinase-9 (MMP-9) were significantly different between ENDS users and non-smokers, while biomarkers of inflammatory mediators, such as MPO, receptor for advanced glycation end products (RAGE), and uteroglobin/CC-10, showed significant differences between dual users and ENDS groups. However, no significant differences in biomarkers of immunity, tissue repair, or growth factors were found between the groups. Similarly, Alqahtani et al.^24^ found that ENDS users had significantly higher levels of pro-inflammatory markers, such as IL-1β and tumour necrosis factor-α (TNF-α), compared to controls, along with distinct metabolite profiles suggesting an increased risk for periodontal disease^24^.

Verma et al.^25^ and Faridoun et al.^26^ further underscored the relationship between ENDS use and elevated levels of proinflammatory cytokines^25,26^. Verma et al.^25^ found significant increases in TNF-α among ENDS users, dual users, and cigarette smokers compared to non-smokers, along with elevated IL-1β and reduced IL-1RA, though the latter two did not reach statistical significance. Likewise, Faridoun et al.^26^ reported increased IL-1β and significantly higher TNF-α levels in ENDS users, indicating an amplified inflammatory response^26^.

Of the five studies investigating the biological impacts of ENDS use, two relied on self-reported data for smoking and ENDS use, and two did not specify their assessment methods. None of the studies used biochemical verification to control for smoking-related confounding. Additionally, Wadia et al.^27^ examined gingival inflammation in smokers who switched to vaping for two weeks, but the observed effects could be related to normal changes in bleeding from smoking cessation, complicating interpretation^27^. Therefore, caution should be applied in interpreting these findings, and it is essential to address potential confounders by accurately controlling for smoking status and history.

### Microbiology

Five studies in this review investigated microbiome changes and found that ENDS use is associated with alterations in the oral microbiome, with specific microbial shifts tied to oral health risks. The collection methods varied across studies, including soft tissue oral swabs, saliva samples, and subgingival plaque samples. Three studies focused on analysing alpha diversity (species diversity within a specific ecosystem) and beta diversity (the similarity or dissimilarity between different communities).

Yang et al.^28^ identified distinct beta diversity differences between vapers and non-vapers, with dual users exhibiting higher alpha diversity and a unique beta diversity^28^. Similarly, Park et al.^29^ observed increased alpha diversity and differences in beta diversity among EC users^29^. Further supporting these findings, Thomas et al.^30^ noted that while ENDS users shared microbial traits with smokers and non-smokers, they had a unique microbiome enriched with specific species^30^. Xu et al.^31^ also noted that ENDS use enriched periodontal disease-associated pathogens like Porphyromonas gingivalis and Fusobacterium nucleatum, with increased levels of proinflammatory cytokines (IL-10, IL-12p70, IL-13 and TNF-α) contributing to microbiome dysbiosis^31^. Ganesan et al.^32^ demonstrated that ENDS trigger inflammation through distinct pathways and promote biofilm growth, altering microbial communities within 24 h^32^.

However, caution must be applied to the findings of these studies. Three studies did not biologically verify smoking status and relied upon self-reporting. One study employed a urine cotinine test as well as a carbon monoxide test, however, did not report the data^29^. Most interestingly, data from a study which utilised a carbon monoxide breath test highlighted its presence in both the ENDS only group and the non-smoking group^30^. This suggests that some participants were current smokers, given the relatively short half-life of carbon monoxide^33^. This undermines the findings by confirming the presence of an important confounding factor.

### Publication geography

The studies included in this review span multiple countries and populations (Fig. S24, supplementary materials). However, a large number were conducted in the United States (US) and Saudi Arabia, with most of the research on the effects of ENDS use on PROMs originating from the US.

### Publication bias

Funnel plots showed asymmetry (Figs. S3, S4, S19, S20, S21, S22, S23, supplementary material), potentially suggesting smaller studies may be missing but this pattern was not consistent.

## Discussion

One of the key findings of this review is that the studies conducted in this field to date are often at high risk of bias with the main issue being that the ENDS group is confounded by tobacco smoking i.e. those individuals classed as ‘ENDS users’ were also smoking tobacco. We viewed the gold standard in this respect to be self-reported status supported by biochemical verification but none of the studies included in our meta-analysis did this. Indeed, some studies explicitly allowed current and/or former smoking in their ENDS groups but often this was simply not considered or reported. Additionally, ENDS use itself was poorly reported or quantified with most studies simply including self-reported ENDS users with no further criteria or thresholds. Hence the other findings of this review should be considered ‘low certainty’ which is probably to be expected given the relatively early nature of the research field (e-cigarettes have only been around for a couple of decades) and the challenges of conducting studies with ENDS users (they are usually current/former smokers).

When considering clinical periodontal outcomes for ENDS users we found them most similar to non-smokers/former smokers with smokers having consistently worse outcomes across different measures.

For the more definitive measures of periodontal destruction, such as PPDs and marginal bone loss, we did not find evidence that ENDS users had poorer outcomes compared to non-smokers/former smokers (smokers had worse outcomes). However, there was evidence that plaque levels were higher in ENDS users compared to non-smokers/former smokers (smokers had worse outcomes) which is in keeping with findings from some in vitro laboratory studies (which reported increased biofilm formation)^34^. Although caries was beyond the scope of this review, this increased plaque would be in keeping with a US study which found an association with caries^35^.

Interestingly the evidence on BOP and gingival indices reported that ENDS users had lower BOP/GI than non-smokers/former smokers and there was no evidence for a difference between smoking and ENDS groups. These suppression effects are well known with smoking, but this finding suggests ENDS use has similar effects on gingival vasculature (probably through the effects of nicotine). Although confounding could account for this finding, those studies we identified at lowest risk of confounding showed some of the greatest differences suggesting this was not the case for this outcome. Interestingly this finding of reduced BOP/GI is contrary to what two pilot studies reported^11,27^.

Studies also indicated that ENDS use leads to unique microbial changes compared to tobacco smokers and higher pro-inflammatory markers compared to non-smokers/former smokers. Large self-reported survey studies did collectively suggest increased gingival/periodontal damage with ENDS use but these studies are again limited by their self-reported nature and the impact of tobacco smoking confounding. Interestingly one of the studies was able to control for tobacco smoking and reported that this accounted for the changes observed^22^.

One of the most important and interesting clinical questions is what happens to the periodontal health when tobacco smokers ‘switch’ to ENDS use (quitting tobacco smoking). The two studies we included that investigated this situation reported either neutral or positive effects (i.e. periodontal health improved), but both these studies have major limitations^12,14^. A large ongoing trial, ENHANCE-D, will help further inform this question^13^.

Overall, these findings on periodontal health are in keeping with much of the public health messaging on ENDS and general health – ENDS use is not risk free but far less harmful than tobacco smoking; “If you smoke, vaping is much safer; if you don’t smoke, don’t vape”^36–39^.

Our systematic review has several strengths. We identified that previous systematic reviews had not assessed the presence of tobacco smoking confounding in any detail and hence we paid particular attention to this in our review, finding it to be a critical issue in the research field. In our meta-analysis we stratified studies by their approach to this which allowed potential impacts to be visualised. Moreover, only two previous reviews (of 6) applied appropriate RoB tools to assess bias, with others either using critical appraisal tools or not assessing bias at all (Table S4, supplementary materials). We used appropriate RoB tools on each of our included studies. Across our analyses we found substantial statistical heterogenicity probably reflecting the variability of the included studies. Subgroup analysis sometimes suggested that tobacco smoking confounding could explain some of the heterogenicity. Overall, our conclusions have been tempered to take this into account.

Some previous reviews also focused on studies from a single geographical location, limiting the generalizability of their findings. For instance, the studies in Figueredo et al.^6^ included a total of 926 male participants and only 11 female participants, with all studies conducted in Saudi Arabia^6^. In contrast, our review includes studies from various countries (Fig. S19, supplementary materials), representing a more global population, though many studies still come from the US and Saudi Arabia, especially those examining the effects of ENDS on PROMs.

**Fig. 5:**
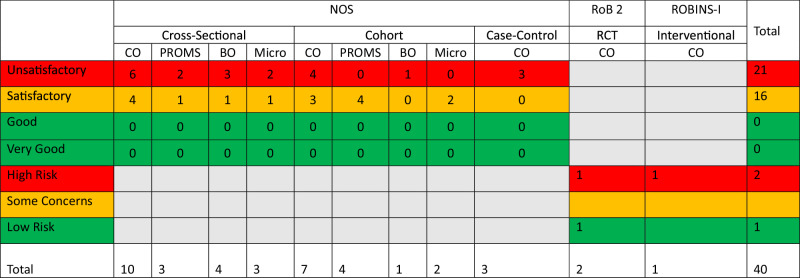
Risk of Bias Summary.

Overall, the available evidence on the impacts of ENDS use on periodontal health is limited and carries a high risk of bias (Fig. 5). Therefore, results from these studies and this review should be interpreted and applied with caution. Randomised controlled trial designs are not always suitable in this field due to ethical issues, leading to a reliance on observational studies to explore these effects. The design and methods of observational studies on ENDS and periodontal health can be enhanced for clearer insights by including tobacco smoking specific biomarkers (not biomarkers of nicotine) where possible to allow better categorisation of the user groups.

## Conclusion

Within the limitations of the included studies, which are at high risk of bias, usually from confounding, we found evidence that ENDS use had some impacts on periodontal parameters compared to non-smokers/former smokers, but tobacco smokers had consistently worst outcomes. Further well-designed research is required in this field to inform clinical practice and guidelines.

## Supplementary information

Supplementary Material

PRISMA Checklist

## Data Availability

All the data used in this study is either available in the supplementary material or at the Newcastle University repository (10.25405/data.ncl.27059734).
