# Supplementary material for: The impact of e-cigarette use on periodontal health: a systematic review and meta-analysis
Source: Evid Based Dent. 2025 Feb 13;26(2):117–8. doi: 10.1038/s41432-025-01119-6 (PMC12204857; doi:10.1038/s41432-025-01119-6)
Supplement: Supplementary file 1 — Supplementary Material [file 41432_2025_1119_MOESM1_ESM.docx]

Supplementary Materials

Contents

[1. Inclusion and Exclusion Criteria 2](#_Toc178366223)

[1.1 Participants/population 2](#_Toc178366224)

[1.2 Intervention(s), exposure(s) 2](#_Toc178366225)

[1.3 Comparator(s)/control 2](#_Toc178366226)

[1.4 Types of study to be included 2](#_Toc178366227)

[2. Full Materials and Methods 3](#_Toc178366228)

[3. Search strategies used on 29/02/2024 6](#_Toc178366229)

[3.1 MEDLINE and EMBASE databases using the OVID platform 6](#_Toc178366230)

[3.3 PubMed 6](#_Toc178366231)

[3.2 Web of Science 6](#_Toc178366232)

[3.4 CINHAL Plus and Dentistry & Oral Sciences Source databases using the EBSCO platform 7](#_Toc178366233)

[4. Data extraction 10](#_Toc178366234)

[5. Table S1. Complete NOS RoB assessments 11](#_Toc178366235)

[6. Table S2. RoB results for studies assessed using the Cochrane RoB 2.0 Tool 15](#_Toc178366236)

[7. Table S3. RoB results for study assessed using the Cochrane ROBINS-I Tool 16](#_Toc178366237)

[8. Additional Forest and Funnel plots (Figures S1 – S18) 17](#_Toc178366238)

[9. Figure S19. Geography of included studies. 40](#_Toc178366239)

[10. Adapted NOS RoB tools 41](#_Toc178366240)

[10.1 NEWCASTLE - OTTAWA QUALITY SCALE ADAPTED FOR CASE CONTROL STUDIES FOR ENDS STUDIES ON PERIODONTAL HEALTH 41](#_Toc178366241)

[10.2 NEWCASTLE - OTTAWA QUALITY SCALE ADAPTED FOR COHORT STUDIES FOR ENDS STUDIES ON PERIODONTAL HEALTH 43](#_Toc178366242)

[10.3 NEWCASTLE-OTTAWA SCALE ADAPTED FOR CROSS-SECTNAL FOR ENDS STUDIES ON PERIODONTAL HEALTH 45](#_Toc178366243)

[11. Table S4. Summary of previous systematic reviews on ENDS use and periodontal health 47](#_Toc178366244)

# 1. Inclusion and Exclusion Criteria

## 1.1 Participants/population

Inclusion: Patients that fall into one of three cohorts: orally healthy, suffering from gingivitis or periodontitis.

Exclusion: Other nicotine products or tobacco devices such as heated tobacco products and nicotine pouches. No additional exclusion criteria pertaining to participants or populations will be imposed, as this approach is likely to constrain the inclusivity of the review. Such limitations may arise from the fact that numerous studies may not provide detailed information regarding any specified exclusion criteria, potentially hindering the comprehensive incorporation of relevant research in the analysis.

## 1.2 Intervention(s), exposure(s)

Exposure: The use of e-cigarettes

## 1.3 Comparator(s)/control

Control: Relevant comparator groups for the study design e.g. Current smokers; former smokers; former smoker, current e-cigarette users; current e-cigarette users; former e-cigarette users.

## 1.4 Types of study to be included

Inclusion: randomized control trials (RCTs), cohort studies, case-control studies, case series, and qualitative studies.

Exclusion: narrative studies, case reports, abstracts, systematic/scope reviews, letters, and expert opinions

# 2. Full Materials and Methods

This systematic review protocol was registered with PROSPERO on March 1, 2024, under ID CRD42024496560. The review adheres to the Preferred Reporting Items for Systematic Review and Meta-Analyses (PRISMA) guidelines (Moher et al. 2009).

**2.1 Eligibility Criteria**

Studies were deemed eligible for inclusion in the review if they involved individuals from one of three cohorts: orally healthy, those with gingivitis, or those with periodontitis. The interventions and comparisons considered were the use of electronic nicotine delivery systems (ENDs) and relevant comparators. Appropriate comparison groups included never smokers (NS), current smokers (CS), dual users of cigarettes and ENDs (DS), former smokers, and former smokers who are current e-cigarette users. The primary outcomes assessed were periodontal parameters such as periodontal probing depths (PPDs), marginal bone loss (MBL), clinical attachment loss (CAL), gingival index (GI), bleeding on probing (BOP), plaque index (PI), and plaque scores. Secondary outcomes included patient-reported outcomes (PROMS), biological biomarkers, and changes in the microbiology of the dental biofilm.

Eligible study designs encompassed observational studies (cross-sectional, case-control, cohort studies), interventional studies (randomized controlled trials, quasi-experimental studies), and qualitative studies. Excluded from this review were review articles, case reports, letters, conference abstracts, narrative studies, systematic/scoping reviews, and expert opinions. Studies included in this review were restricted to human subjects and publications in the English language.

**2.2 Search methods**

Preliminary electronic searches were conducted to aid in the development of the electronic search strategy. This strategy employed a combination of controlled vocabulary (MeSH) and free text terms, which was then piloted to ensure high sensitivity over high precision, maintaining a broad scoping search. The search strategy used consistent terminology tailored to each database, with a restriction to English-language articles. Electronic databases searched up to February 29, 2024, with no year restrictions, included PubMed/MEDLINE, Embase, Web of Science, CINAHL Plus, and Dentistry & Oral Sciences Source (see Search Strategies in supplementary materials). Additionally, hand-searching of bibliographies from previously published reviews was conducted. The search results from all databases were combined, and duplicates were removed.

**2.3 Study selection**

Titles and abstracts of all identified reports were independently screened by two reviewers (RT and JJ) using the predefined inclusion/exclusion criteria. For studies that appeared to meet the inclusion criteria or lacked sufficient information in the title or abstract to confirm eligibility, full-text reports were obtained and assessed independently and in duplicate. Any discrepancies following the full-text screening were resolved through discussion, and an additional reviewer (RH) was consulted. Covidence systematic review software, Veritas Health Innovation, Melbourne, Australia (available at www.covidence.org) was used to record the decision to include or exclude each article.

**2.4 Data extraction**

The clinical, biological, and microbiology data extraction forms were adapted from established systematic review methodologies (Nath et al. 2022; Gupta et al. 2018; Fábio et al. 2018). For PROMS data, this review incorporates diverse datasets and surveys, notably from the PATH survey. A standardised data extraction form was developed in Excel spreadsheets. Data extraction forms were piloted to extract relevant information from included studies for clinical outcomes, biological outcomes, PROMS, and microbiology. Two reviewers (RT and JJ) extracted the data. Information on study characteristics, including population, interventions, comparisons, and types of outcomes reported, was transferred into evidence tables to summarise the included studies and available data. All entries in the Excel spreadsheets were reviewed to assess their suitability for meta-analysis. Finally, the clinical outcomes data was entered into RevMan Web (Version 8.4.1, 27.08.24) in preparation for quantitative analysis.

**2.5 Outcome measures**

Baseline outcomes were extracted from all included studies. The primary outcomes encompassed changes in periodontal parameters which included PPD, MBL, CAL, GI, BOP, PI, and plaque scores. Secondary outcomes included PROMS, and changes in the microbiological profile of the dental biofilm, as well as levels of biological biomarkers in the saliva and/or gingival crevicular fluid (GCF).

**2.6 Risk of bias assessment**

Risk of bias (RoB) assessment for all included studies was conducted independently and in duplicate by two reviewers (RT and JJ) during data extraction. RoB was evaluated using the Cochrane Risk of Bias in Non-randomised Studies - of Interventions (ROBINS-I) tool for interventional studies and the RoB 2 tool for randomized controlled trials. The Cochrane ROBINS-I tool was only used for interventional studies where the intervention was smoking cessation and/or e-cigarette use among current smokers. For observational studies, the Newcastle-Ottawa Scale (NOS) was used and adapted, following previously published methodologies (Herzog et al. 2013), to better suit cross-sectional studies and studies investigating the effects of ENDS use on periodontal health (see Adapted NOS RoB Tools in supplementary materials). Observational studies were evaluated on sample representativeness, the appropriateness of exposure and outcome measurements, control of confounding variables, and clarity of reporting. Cross-sectional and case-control studies were assigned a score ranging from 0 to 10 stars, with higher scores indicating a lower risk of bias. Cohort studies were scored out of 11 stars to account for an additional star assigned to outcomes, allowing for appropriate weighting and differentiation between validated/unvalidated and blinded/unblinded methods. The scoring range for cohort studies was also adjusted accordingly to reflect this extra star.

**2.7 Data synthesis and analysis**

We conducted our analyses in RevMan Web (Version 8.4.1, 27.08.24). Where possible, we pooled studies that reported clinical outcomes using an inverse variance random‐effects model to generate pooled mean differences (MD) or standard mean differences (SMD; if different indices were used to assess the same outcome) with 95% confidence intervals (CIs). Separate analyses were completed for cross-sectional data (including baseline values from intervention studies) and post-intervention data, where data allowed, for each outcome measure. Separate analyses were also completed for two comparisons: ENDS verses non-smokers/former smokers and ENDS verses smokers. We used subgroup analysis to explore the impact of ENDS group confounding risk (i.e. the risk of confounding from current or previous tobacco smoking) using three groups: studies with biochemical verification of ENDS users, studies who relied on self-reporting of ENDS use which were at lower risk of confounding (current smoking not permitted; former smoking not permitted or not mentioned in methods) and studies who relied on self-reporting of ENDs use which were at higher risk of confounding (current and former smokers explicitly allowed). This approach was different to our pre-published protocol as the included studies did not give sufficient detail to facilitate our original groups. To evaluate the heterogeneity of the included studies, the I^2^ statistic was calculated. Funnel plots were used to assess reporting bias in meta-analysed outcome measures that included at least 10 studies.

# 3. Search strategies used on 29/02/2024

## 3.1 MEDLINE and EMBASE databases using the OVID platform

The search strategy detailed below will be used to search the MEDLINE and EMBASE databases using the OVID platform:

1. exp *vaping/

2. *nicotine/

3. exp *electronic cigarette/ or exp smoking device/

4. Electronic Nicotine Delivery Systems.mp.

5. exp *periodontitis/ or exp mouth inflammation/ or exp *periodontal disease/ or exp aggressive periodontitis/ or exp chronic periodontitis/ or exp periodontal pocket/

6. exp *periodontal parameters/ or exp *bleeding on probing/ or exp *clinical attachment level/ or exp *gingival bleeding index/ or exp gingival index/ or exp periodontal index/ or exp *periodontal pocket depth/ or exp *plaque index/ or exp sulcus bleeding index/

7. exp *alveolar bone loss/

8. exp *gingivitis/ or exp *gingiva disease/

9. 1 or 2 or 3 or 4

10. 5 or 6 or 7 or 8

11. 9 and 10

12. limit 11 to (human and english language)

13. remove duplicates from 12

## 3.3 PubMed

((((e-cigarettes) OR (Electronic Nicotine Delivery Systems)) OR (nicotine)) OR (vaping)) AND (((((((Periodontal probing depth) OR (Periodontal pocket depth)) OR (Plaque index)) OR ("Bleeding on probing")) OR (Clinical Attachment Loss)) OR (Periodontal pocket)) OR (periodontitis))

## 3.2 Web of Science

# Web of Science Search Strategy (v0.1)

Search: ALL=(e-cigarettes) Results: 10096

Search: ALL=(Electronic Nicotine Delivery Systems) Results: 1984

Search: ALL=(nicotine) Results: 69264

Search: ALL=(vaping) Results: 4348

Search: #1 OR #2 OR #3 OR #4 Results: 75442

Search: ALL=(Periodontal probing depth) Results: 7307

Search: ALL=(Periodontal pocket depth) Results: 4482

Search: ALL=(Plaque index) Results: 16923

Search: ALL=(Bleeding on probing) Results: 6187

Search: ALL=(Clinical Attachment Loss ) Results: 5719

Search: ALL=(Periodontal pocket ) Results: 7479

Search: ALL=(periodontitis) Results: 39562

Search: #6 OR #7 OR #8 OR #9 OR #10 OR #11 OR #12 Results: 61211

# Database: Web of Science Core Collection

# Entitlements:

- WOS.IC: 1993 to 2024

- WOS.CCR: 1985 to 2024

- WOS.SCI: 1900 to 2024

- WOS.AHCI: 1975 to 2024

- WOS.BHCI: 2005 to 2024

- WOS.BSCI: 2005 to 2024

- WOS.ESCI: 2015 to 2024

- WOS.ISTP: 1990 to 2024

- WOS.SSCI: 1900 to 2024

- WOS.ISSHP: 1990 to 2024

# Searches:

Search: #5 AND #13 and English (Languages) and Review Article or Letter or Editorial Material

or Meeting Abstract or Book Chapters (Exclude – Document Types) Results: 289

## 3.4 CINHAL Plus and Dentistry & Oral Sciences Source databases using the EBSCO platform

| **#** | **Query** | **Limiters/Expanders** | **Last Run Via** | **Results** |
| --- | --- | --- | --- | --- |
| S15 | ((S12) OR (S5 OR S6 OR S7 OR S8 OR S9 OR S10 OR S11 OR S12)) AND (S4 AND S13) | Limiters - English Language; Human Expanders - Apply equivalent subjects Search modes - Boolean/Phrase | Interface - EBSCOhost Research Databases Search Screen - Advanced Search Database - CINAHL Plus;Dentistry & Oral Sciences Source | 314 |
| S14 | ((S12) OR (S5 OR S6 OR S7 OR S8 OR S9 OR S10 OR S11 OR S12)) AND (S4 AND S13) | Expanders - Apply equivalent subjects Search modes - Boolean/Phrase | Interface - EBSCOhost Research Databases Search Screen - Advanced Search Database - CINAHL Plus;Dentistry & Oral Sciences Source | 374 |
| S13 | (S12) OR (S5 OR S6 OR S7 OR S8 OR S9 OR S10 OR S11 OR S12) | Expanders - Apply equivalent subjects Search modes - Boolean/Phrase | Interface - EBSCOhost Research Databases Search Screen - Advanced Search Database - CINAHL Plus;Dentistry & Oral Sciences Source | 68,876 |
| S12 | gingivitis or gum disease or gum inflammation | Expanders - Apply equivalent subjects Search modes - Boolean/Phrase | Interface - EBSCOhost Research Databases Search Screen - Advanced Search Database - CINAHL Plus;Dentistry & Oral Sciences Source | 12,969 |
| S11 | periodontal pocket depth | Expanders - Apply equivalent subjects Search modes - Boolean/Phrase | Interface - EBSCOhost Research Databases Search Screen - Advanced Search Database - CINAHL Plus;Dentistry & Oral Sciences Source | 960 |
| S10 | alveolar bone loss | Expanders - Apply equivalent subjects Search modes - Boolean/Phrase | Interface - EBSCOhost Research Databases Search Screen - Advanced Search Database - CINAHL Plus;Dentistry & Oral Sciences Source | 3,857 |
| S9 | plaque index | Expanders - Apply equivalent subjects Search modes - Boolean/Phrase | Interface - EBSCOhost Research Databases Search Screen - Advanced Search Database - CINAHL Plus;Dentistry & Oral Sciences Source | 7,752 |
| S8 | periodontal parameters | Expanders - Apply equivalent subjects Search modes - Boolean/Phrase | Interface - EBSCOhost Research Databases Search Screen - Advanced Search Database - CINAHL Plus;Dentistry & Oral Sciences Source | 3,077 |
| S7 | clinical attachment level | Expanders - Apply equivalent subjects Search modes - Boolean/Phrase | Interface - EBSCOhost Research Databases Search Screen - Advanced Search Database - CINAHL Plus;Dentistry & Oral Sciences Source | 4,531 |
| S6 | bleeding on probing | Expanders - Apply equivalent subjects Search modes - Boolean/Phrase | Interface - EBSCOhost Research Databases Search Screen - Advanced Search Database - CINAHL Plus;Dentistry & Oral Sciences Source | 6,792 |
| S5 | periodontitis or periodontal disease | Expanders - Apply equivalent subjects Search modes - Boolean/Phrase | Interface - EBSCOhost Research Databases Search Screen - Advanced Search Database - CINAHL Plus;Dentistry & Oral Sciences Source | 54,448 |
| S4 | S1 OR S2 OR S3 | Expanders - Apply equivalent subjects Search modes - Boolean/Phrase | Interface - EBSCOhost Research Databases Search Screen - Advanced Search Database - CINAHL Plus;Dentistry & Oral Sciences Source | 20,638 |
| S3 | electronic nicotine delivery systems | Expanders - Apply equivalent subjects Search modes - Boolean/Phrase | Interface - EBSCOhost Research Databases Search Screen - Advanced Search Database - CINAHL Plus;Dentistry & Oral Sciences Source | 3,406 |
| S2 | nicotine | Expanders - Apply equivalent subjects Search modes - Boolean/Phrase | Interface - EBSCOhost Research Databases Search Screen - Advanced Search Database - CINAHL Plus;Dentistry & Oral Sciences Source | 15,323 |
| S1 | vaping or electronic cigarettes or e-cigarettes or juul or e-cigs | Expanders - Apply equivalent subjects Search modes - Boolean/Phrase | Interface - EBSCOhost Research Databases Search Screen - Advanced Search Database - CINAHL Plus;Dentistry & Oral Sciences Source | 8,073 |

# 4. Data extraction

The size of the data extraction tables precluded their inclusion in the main manuscript and supplementary materials. However, the complete dataset is available in full at the Newcastle University repository (<https://doi.org/10.25405/data.ncl.27059734>).

# 5. Table S1. Complete NOS RoB assessments

| **Category** | **Type of Study** | **RoB tool** | **Study** | **Selection** | | | | **Comparability** | **Outcome / Exposure** | | | **Total** | **Outcome** | |
| --- | --- | --- | --- | --- | --- | --- | --- | --- | --- | --- | --- | --- | --- | --- |
|  |  |  |  | **1** | **2** | **3** | **4** |  | **1** | **2** | **3** |  |  |  |
| Biological Outcomes | Cross Sectional | Cross Sectional NOS | Ye et al. 2020 | C | A* | C | B* | B | D** | A* | - | 5 | Satisfactory |  |
|  | Cross Sectional ^1^ | Cross Sectional NOS | Verma et al. 2021 | C | B | C | B* | B | D** | A* | - | 4 | Unsatisfactory |  |
|  | Cross Sectional ^1^ | Cross Sectional NOS | Alqahtani et al. 2020 | C | B | C | B* | B | D** | A* | - | 4 | Unsatisfactory |  |
|  | Cross Sectional | Cross Sectional NOS | Faridoun et al. 2020 | C | B | C | C | B | D** | A* | - | 3 | Unsatisfactory |  |
|  | Prospective Cohort ^1^ | Cohort NOS | Wadia et al. 2016 | C | C | C | A* | B | D** | B | B* | 4 | Unsatisfactory |  |
| Clinical Outcomes | Prospective Cohort ^1^ | Cohort NOS | Ismail et al. 2019 | C | C | C | A* | B | A* | A* | C | 3 | Unsatisfactory |  |
|  | Retrospective Cohort ^1^ | Cohort NOS | Shah et al. 2022 | C | A* | B* | A* | B | C* | B | D | 3 | Unsatisfactory |  |
|  | Randomized Control Trial | RoB 2 | Alshibani et al. 2022 |  |  |  |  |  |  |  |  |  |  |  |
|  | Cross Sectional | Cross Sectional NOS | Alqahtani et al. 2022 | C | B | C | C | B | C* | A* | - | 2 | Unsatisfactory |  |
|  | Prospective Cohort ^1^ | Cohort NOS | Amaliya et al. 2023 | C | A* | B* | A* | B | C* | B | A* | 5 | Unsatisfactory |  |
|  | Prospective Cohort ^1^ | Cohort NOS | ALHarthi et al. 2019 | C | A* | B* | A* | B | A** | A* | A* | 7 | Satisfactory |  |
|  | Prospective Cohort ^1^ | Cohort NOS | Xu et al. 2021 | C | A* | A** | A* | B | C* | A* | B* | 7 | Satisfactory |  |
|  | Cross Sectional ^1^ | Cross Sectional NOS | Javed et al. 2017 | C | B | C | B* | B | A** | A* | - | 4 | Unsatisfactory |  |
|  | Feasibility with imbedded RCT | ROB 2 | Holiday et al. 2019 |  |  |  |  |  |  |  |  |  |  |  |
|  | Case-Control ^3^ | Case-Control NOS | Ghazali et al. 2018 | C | B | C | B | B | C | B | C | 0 | Unsatisfactory |  |
|  | Prospective Cohort ^1^ | ROBINS-I ^2^ | Tatullo et al. 2016 |  |  |  |  |  |  |  |  |  |  |  |
|  | Cross Sectional | Cross Sectional NOS | Mokeema et al. 2018 | C | A* | C | B* | B | A** | A* | - | 5 | Satisfactory |  |
|  | Case-Control ^3^ | Case-Control NOS | Ali et al. 2022 | C | B | C | B | B | B* | B | C | 1 | Unsatisfactory |  |
|  | Prospective Cohort ^1^ | Cohort NOS | Akram et al. 2021 | C | A* | C | A* | B | D** | A* | D | 5 | Unsatisfactory |  |
|  | Cross Sectional | Cross Sectional NOS | BinShabaiba et al. 2019 | C | B | C | B* | B | D** | A* | - | 4 | Unsatisfactory |  |
|  | Prospective Cohort ^1^ | Cohort NOS | Alhumaidan et al. 2022 | C | A* | B* | A* | B | A** | A* | A* | 7 | Satisfactory |  |
|  | Cross Sectional ^4^ | Cross Sectional NOS | Hamoudi et al. 2020 | C | A* | C | B* | B | D** | A* | - | 5 | Satisfactory |  |
|  | Cross Sectional ^1^ | Cross Sectional NOS | Karaaslan et al. 2020 | C | A* | C | B* | B | A** | A* | - | 5 | Satisfactory |  |
|  | Cross Sectional | Cross Sectional NOS | Vohra et al. 2020 | C | B | C | B* | B | C* | A* | - | 3 | Unsatisfactory |  |
|  | Cross Sectional | Cross Sectional NOS | Aldakheela et al. 2020 | C | B | C | B* | B | C* | A* | ` | 3 | Unsatisfactory |  |
|  | Case-Control ^3^ | Case-Control NOS | Ibraheema et al. 2020 | C | B | C | B | B | B* | B | C | 1 | Unsatisfactory |  |
|  | Cross Sectional ^1^ | Cross Sectional NOS | Pushalkar et al. 2020 | C | B | C | A** | B | D** | A* | - | 7 | Satisfactory |  |
|  | Cross Sectional ^1^ | Cross Sectional NOS | Zieba et al. 2024 | C | A* | C | C | B | D** | A* | - | 4 | Unsatisfactory |  |
| Microbiology | | Cross-sectional | Cross Sectional NOS | Yang et al. 2023 | C | B | C | B* | B | D** | A* | - | 4 | Unsatisfactory |
|  |  | Cross-sectional ^1^ | Cross Sectional NOS | Park et al. 2023 | C | B | C | B* | B | D** | A* | - | 4 | Unsatisfactory |
|  |  | Prospective Cohort ^1^ | Cohort NOS | Thomas et al. 2022 | C | A* | A** | A* | B | D** | A* | D | 7 | Satisfactory |
|  |  | Prospective Cohort ^1^ | Cohort NOS | Xu et al. 2021 | C | A* | C | A* | B | D** | A* | B* | 6 | Satisfactory |
|  |  | Cross-sectional | Cross Sectional NOS | Ganesan et al. 2020 | C | A* | C | B* | B | D** | A* | - | 5 | Satisfactory |
| PROMS | | Prospective Cohort ^1^ | Cohort NOS | Mohajeri et al. 2024 | A* | A* | B* | B | B | F** | A* | B* | 7 | Satisfactory |
|  |  | Prospective Cohort ^1^ | Cohort NOS | Atuegwu et al. 2019 | A* | A* | B* | B | B | F** | A* | B* | 7 | Satisfactory |
|  |  | Cross-sectional | Cross Sectional NOS | Jeong et al. 2019 | C | B | A* | B* | B | G* | A* | - | 4 | Unsatisfactory |
|  |  | Cross-sectional | Cross Sectional NOS | AlQobaly et al. 2022 | C | C | A* | B* | B | G* | A* | - | 4 | Unsatisfactory |
|  |  | Prospective Cohort | Cohort NOS | Wiernik et al. 2024 | A* | A* | B* | B | B | F** | A* | B* | 7 | Satisfactory |
|  |  | Prospective Cohort | Cohort NOS | Silveira et al. 2022 | A* | A* | B* | B | B | F** | A* | B* | 7 | Satisfactory |
|  |  | Cross-sectional | Cross Sectional NOS | Vora & Chaffee 2019 | A* | B | A* | B* | B | F** | A* | - | 6 | Satisfactory |

1. The study designs were not clearly defined by the original authors and are often ambiguous. As a result, these studies have been assessed using the appropriate NOS tools based on the study design their methodologies most closely resemble, as determined by a full-text review by the authors of this review.
2. The Cochrane ROBINS-I RoB tool was used for studies where the intervention was smoking cessation and/or e-cigarette use among current smokers.
3. Although these studies are described as case-control, they lack a clear distinction between cases (those with periodontal disease) and controls (those with healthy periodontal parameters). With no proper control group and simultaneous assessment of exposure and outcome, they resemble cross-sectional studies rather than true case-control designs. However, since the authors describe them as case-control, they have been evaluated accordingly.
4. The study is described as cross-sectional, though its design more closely resembles a prospective cohort study. However, as the authors describe it as cross-sectional, it has been evaluated accordingly.

# 6. Table S2. RoB results for studies assessed using the Cochrane RoB 2.0 Tool


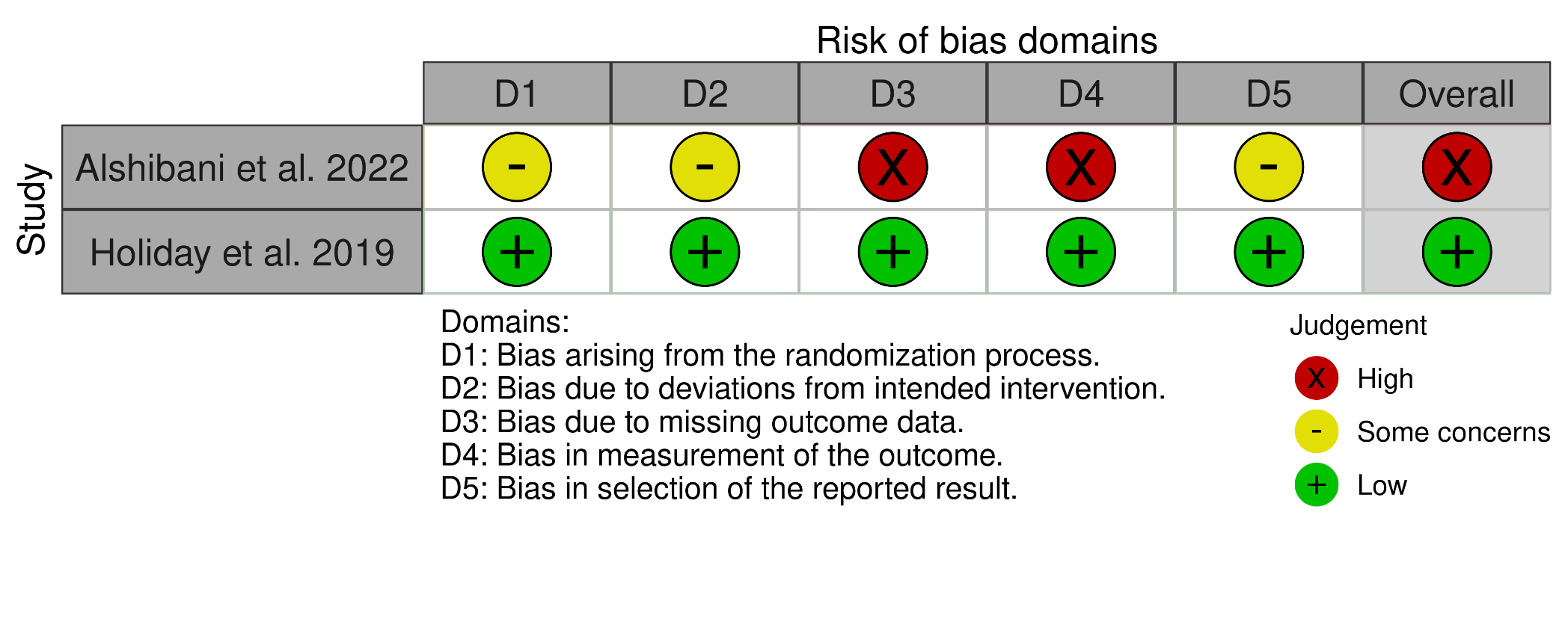


# 7. Table S3. RoB results for study assessed using the Cochrane ROBINS-I Tool


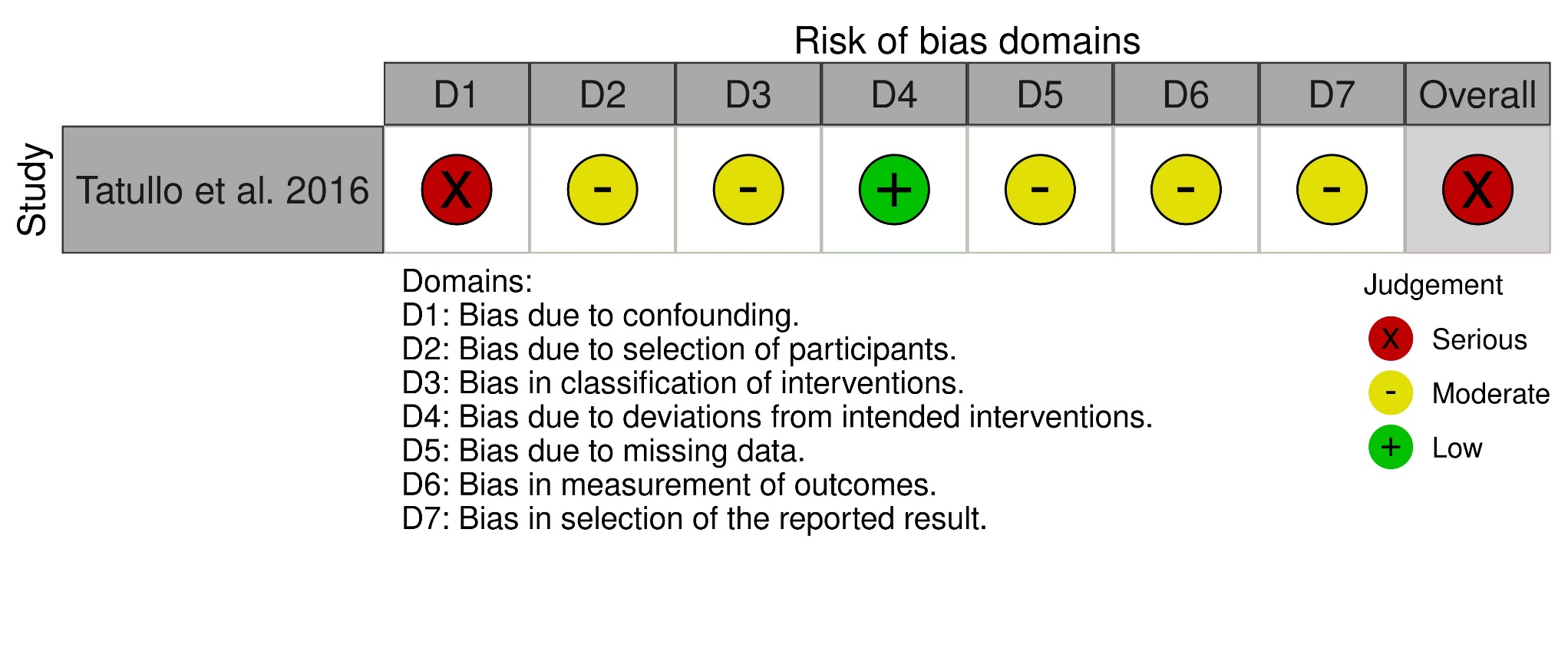


# 8. Additional Forest and Funnel plots (Figures S1 – S23)

Figure S1. Forest plot of Pocket Probing Depth (PPD) comparison for ENDS users verses non-smokers/former smokers for post-intervention data: sub-grouped by risk of tobacco smoking confounding in ENDS group.


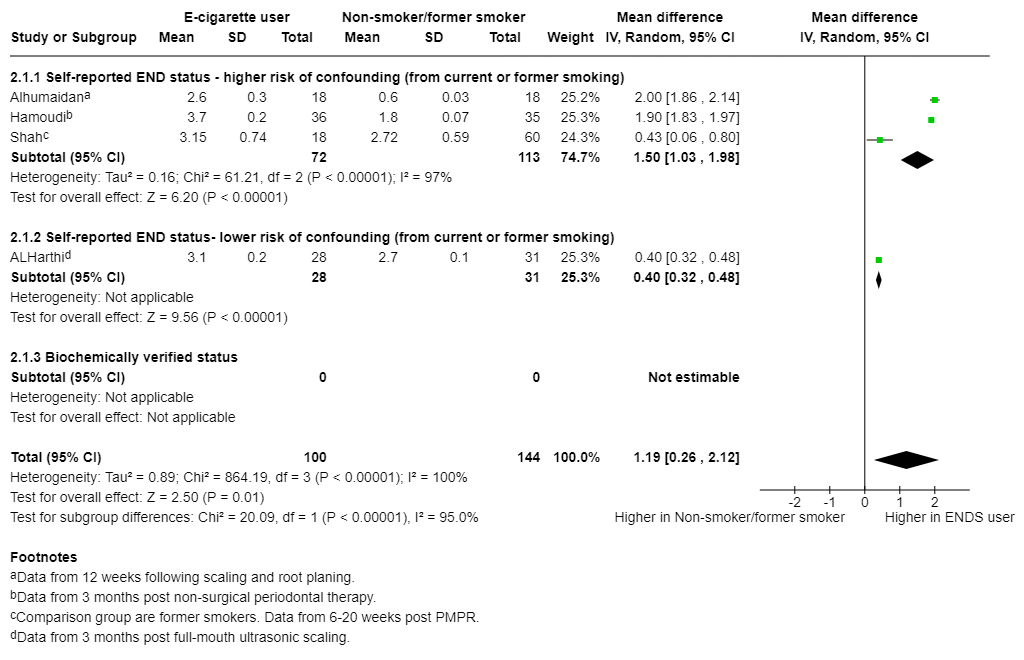


Figure S2. Forest plot of Pocket Probing Depth (PPD) comparison for ENDS users verses smokers for post intervention data: sub-grouped by risk of tobacco smoking confounding in ENDS group.


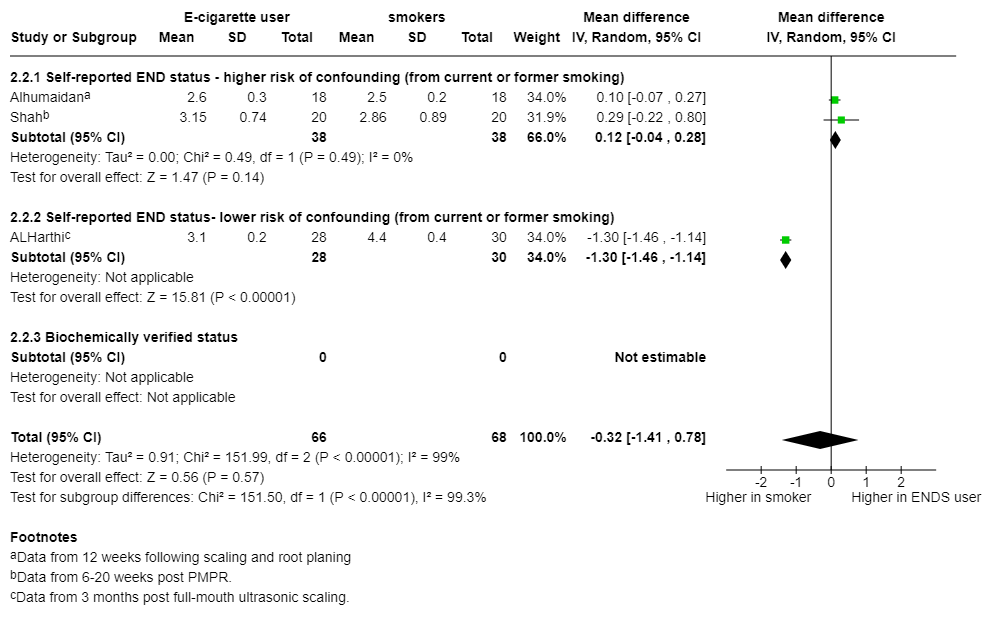


Figure S3. Funnel plot of PPD analysis: ENDS verses non-smoker/former smoker (cross-sectional data) from Figure 2A.


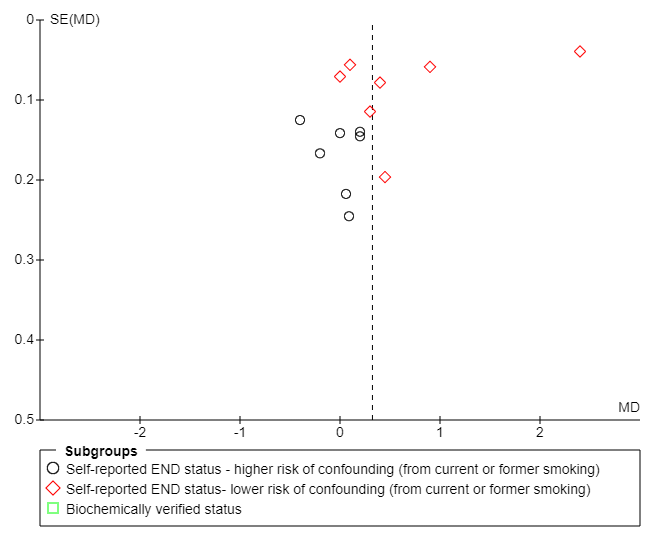


Figure S4. Funnel plot of PPD analysis: ENDS verses smokers (cross-sectional data) from Figure 2B.


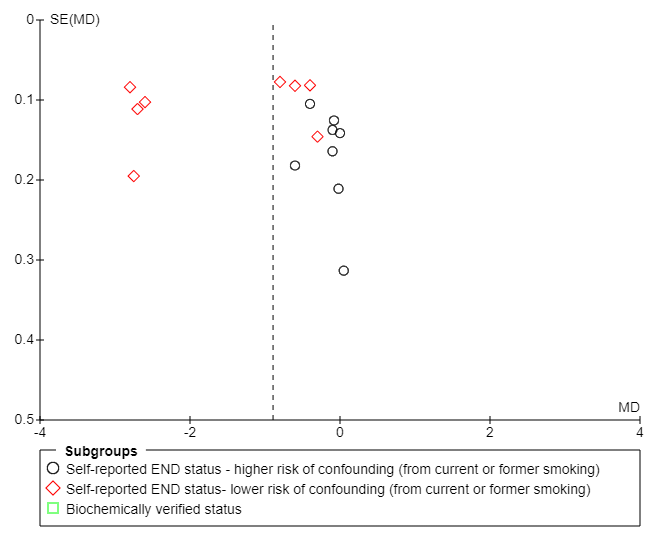


Figure S5. Forest plot of Clinical Attachment Loss (CAL) comparison for ENDS users verses non-smokers/former smokers for cross-sectional data: sub-grouped by risk of tobacco smoking confounding in ENDS group. SMDs were used in the analysis of CAL due to inconsistencies in how CAL was defined and calculated across studies, with varied terms like clinical attachment level, clinical attachment loss, and attachment loss.


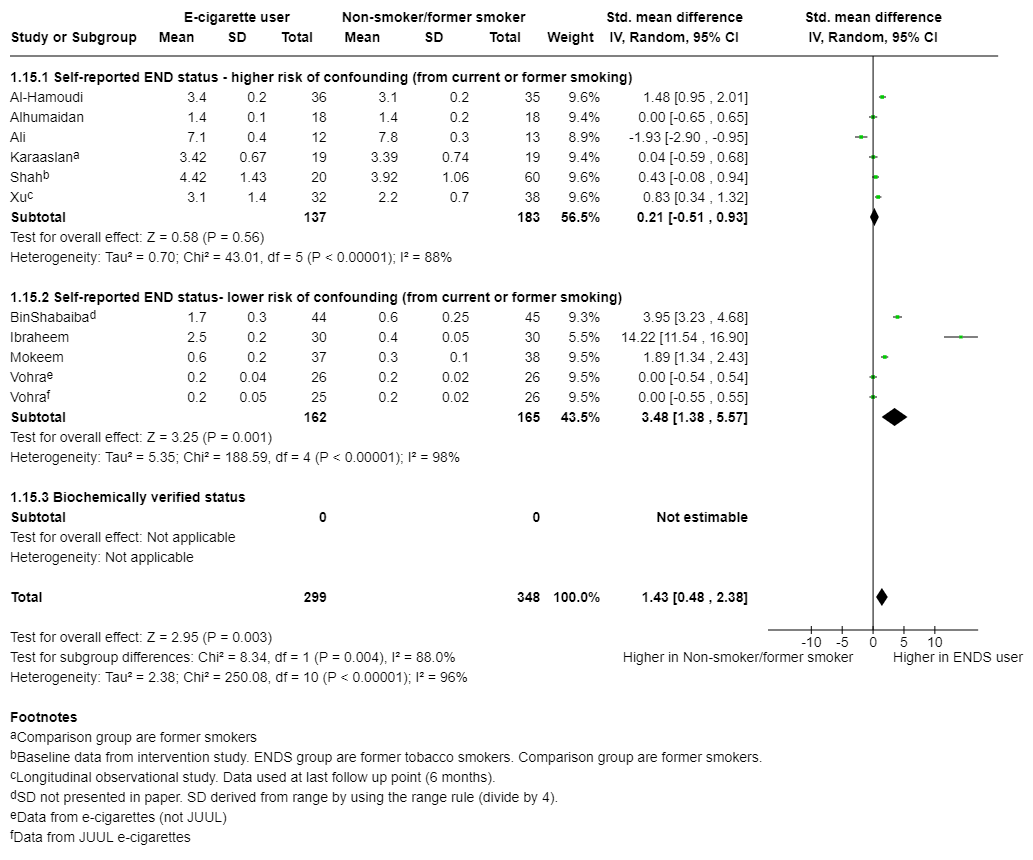


Figure S6. Forest plot of Clinical Attachment Loss (CAL) comparison for ENDS users verses smokers for cross-sectional data: sub-grouped by risk of tobacco smoking confounding in ENDS group. SMDs were used in the analysis of CAL due to inconsistencies in how CAL was defined and calculated across studies, with varied terms like clinical attachment level, clinical attachment loss, and attachment loss.


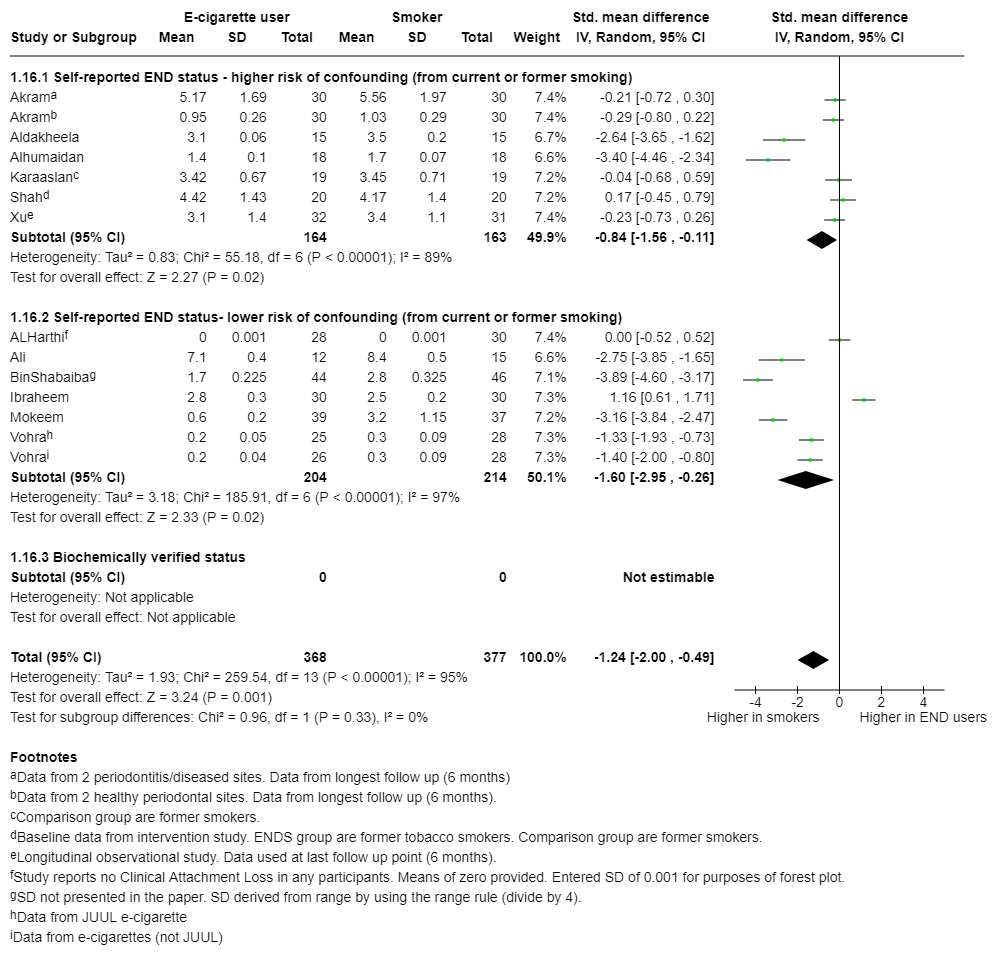


Figure S7. Forest plot of Distal Marginal Bone Loss (MBL) comparison for ENDS users verses non-smokers/former smokers for cross sectional data: sub-grouped by risk of tobacco smoking confounding in ENDS group.


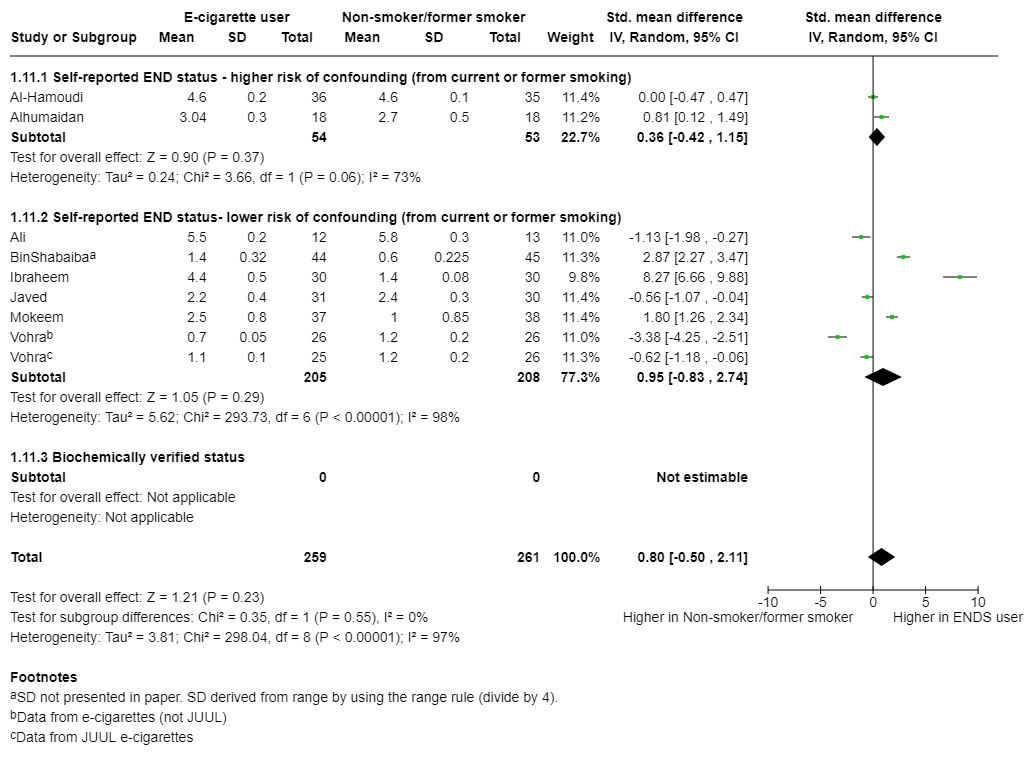


Figure S8. Forest plot of Mesial Marginal Bone Loss (MBL) comparison for comparison for ENDS users verses non-smokers/former smokers for cross-sectional data: sub-grouped by risk of tobacco smoking confounding in ENDS group.


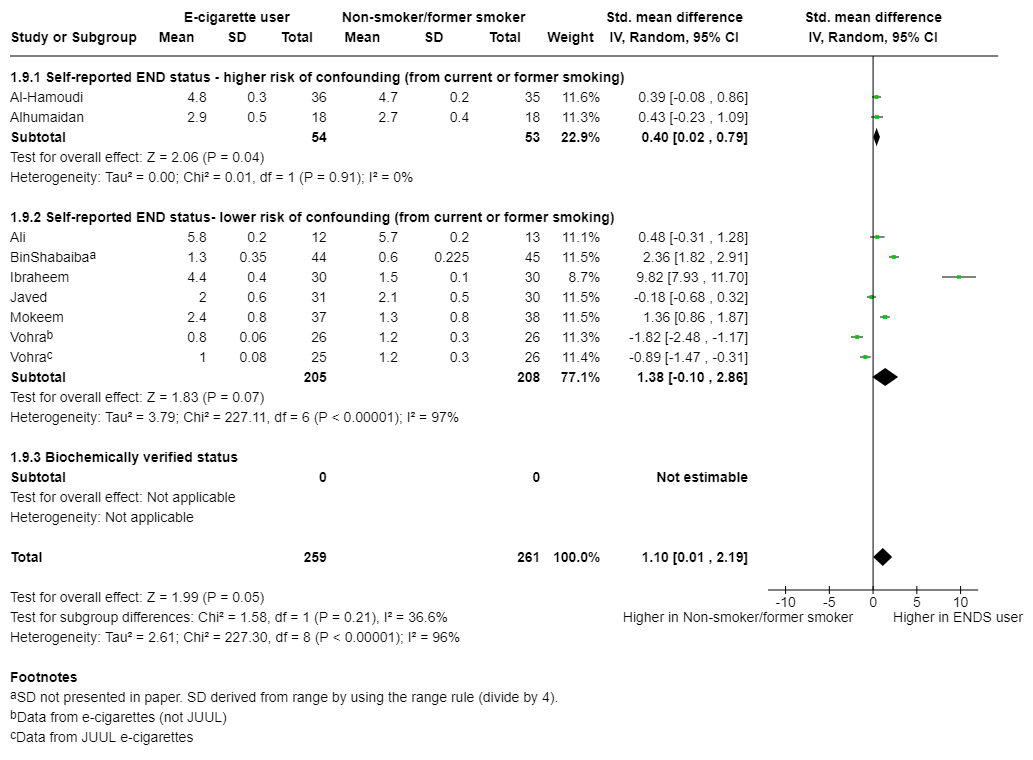


Figure S9. Forest plot of Mesial Marginal Bone Loss (MBL) comparison for ENDS users verses smokers for cross-sectional data: sub-grouped by risk of tobacco smoking confounding in ENDS group.


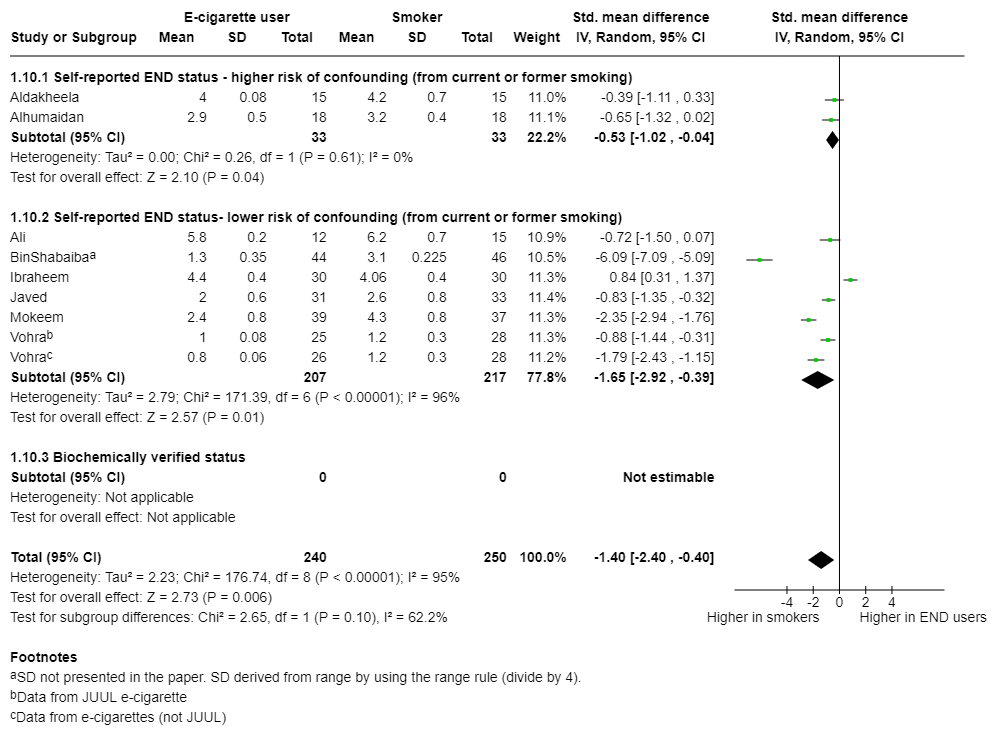


Figure S10. Forest plot of Distal Marginal Bone Loss (MBL) comparison for ENDS users verses smokers for cross-sectional data: sub-grouped by risk of tobacco smoking confounding in ENDS group.


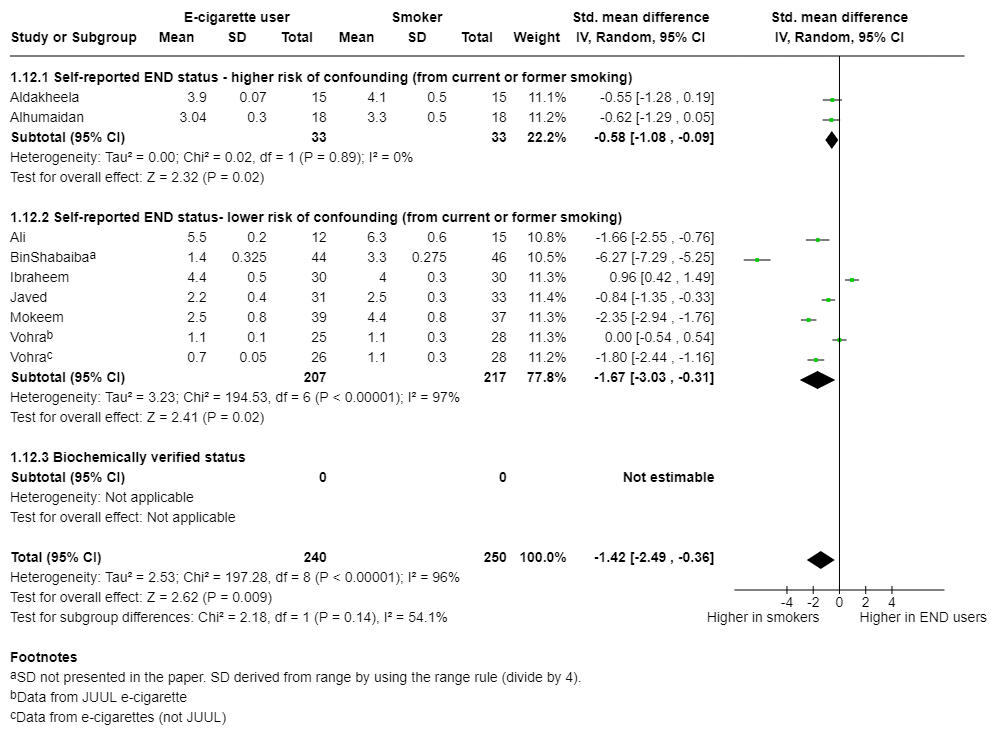


Figure S11. Forest plot of Gingival Indices (GI) comparison for comparison for ENDS users verses non-smokers/former smokers for cross-sectional data: sub-grouped by risk of tobacco smoking confounding in ENDS group.


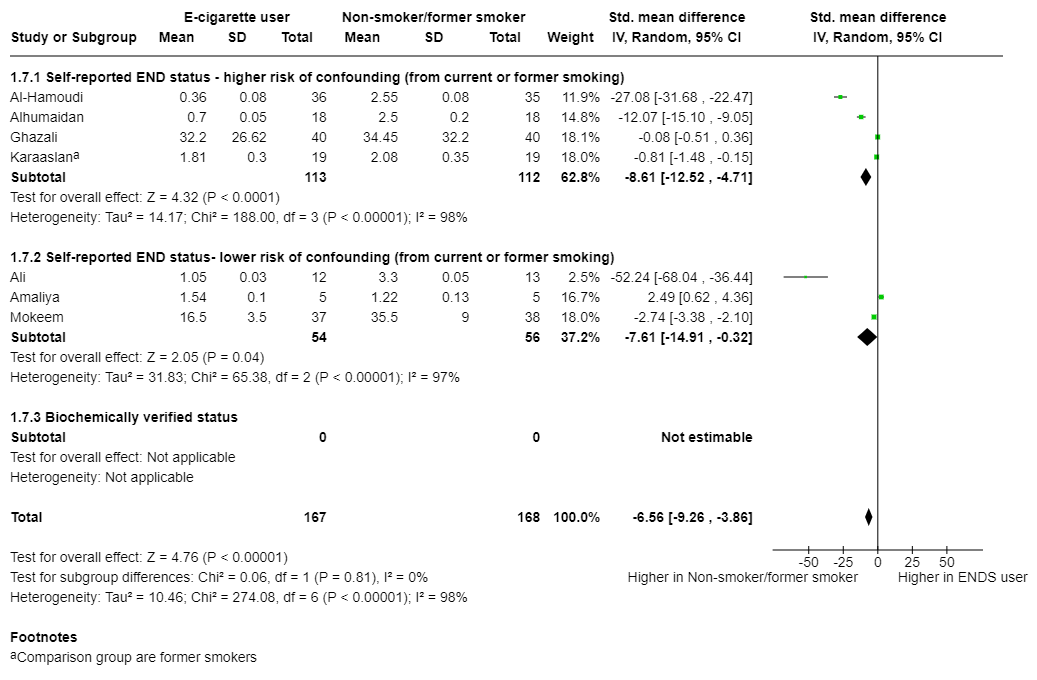


Figure S12. Forest plot of Gingival Indices (GI) comparison for comparison for ENDS users verses smokers for cross-sectional data: sub-grouped by risk of tobacco smoking confounding in ENDS group.


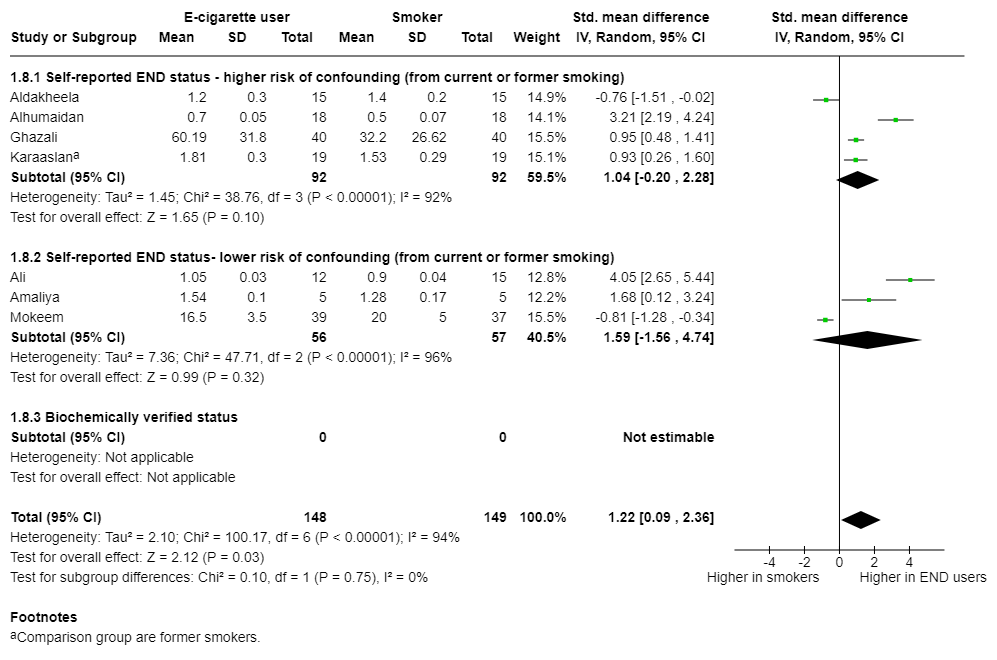


Figure S13. Forest plot of Bleeding on Probing (BOP) comparison for ENDS users verses non-smokers/former smokers for post-intervention data: sub-grouped by risk of tobacco smoking confounding in ENDS group.


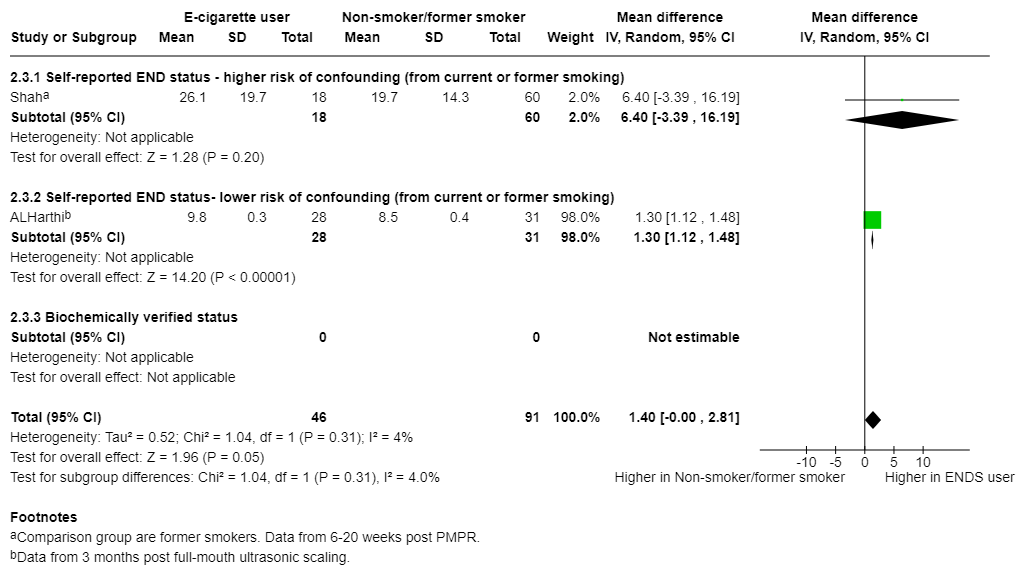


Figure S14. Forest plot of Bleeding on Probing (BOP) comparison for ENDS users verses smokers for post-intervention data: sub-grouped by risk of tobacco smoking confounding in ENDS group.


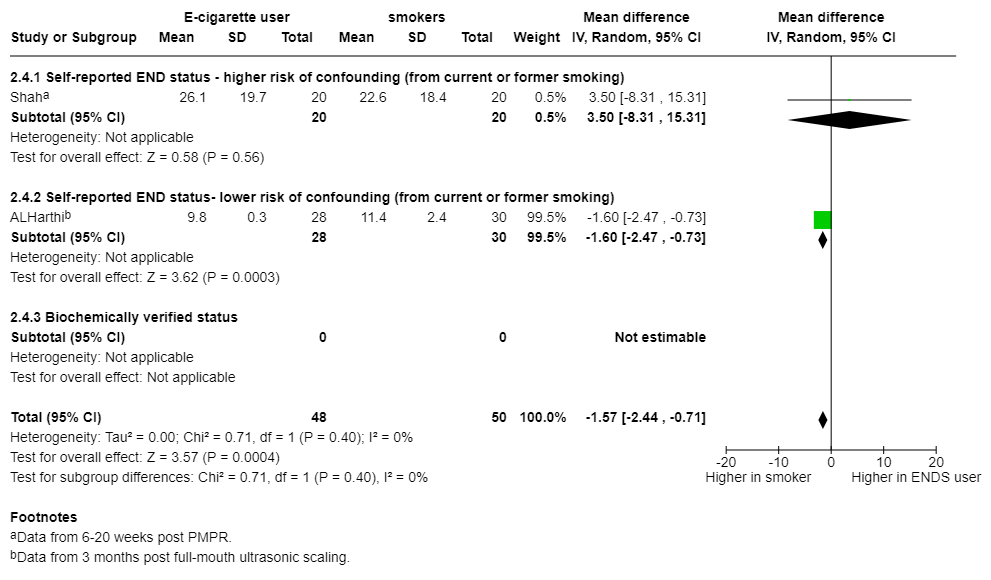


Figure S15. Forest plot of Gingival Indices (GI) comparison for ENDS users verses non-smokers/former smokers for post-intervention data: sub-grouped by risk of tobacco smoking confounding in ENDS group.


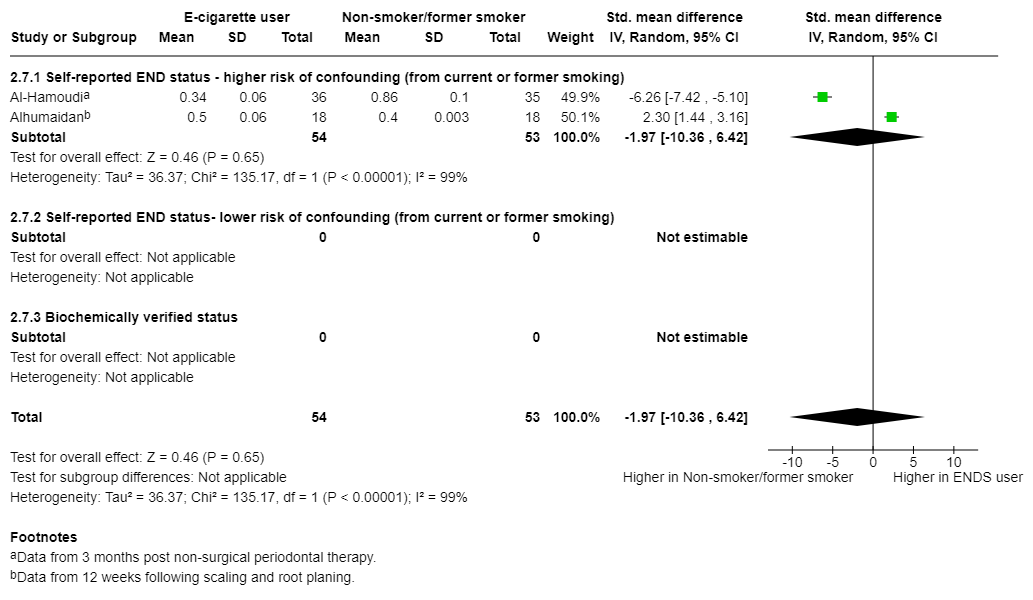


Figure S16. Forest plot of Gingival Indices (GI) comparison for ENDS users verses smokers for post-intervention data: sub-grouped by risk of tobacco smoking confounding in ENDS group.


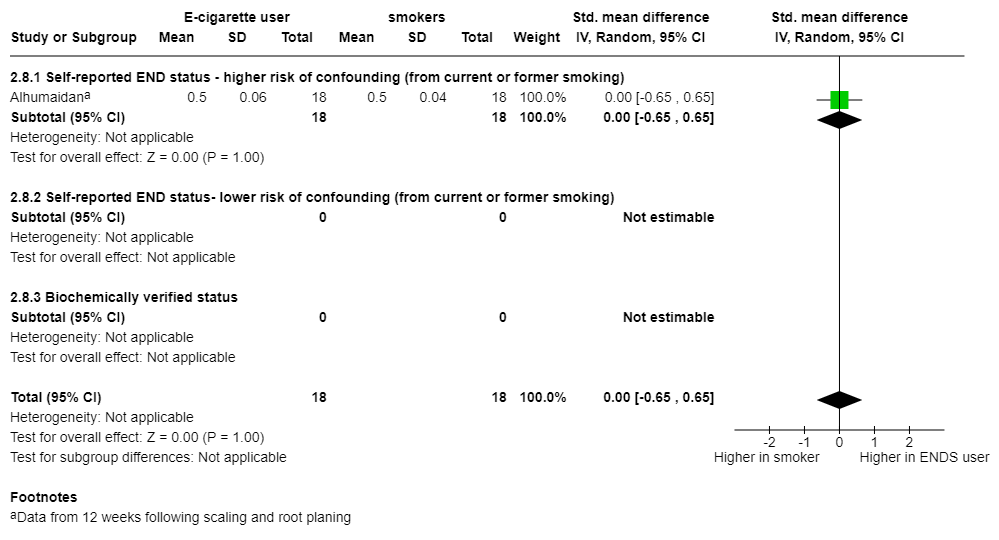


Figure S17. Forest plot of Plaque Index (PI) comparison for ENDS users verses non-smokers/former smokers for post-intervention data: sub-grouped by risk of tobacco smoking confounding in ENDS group.


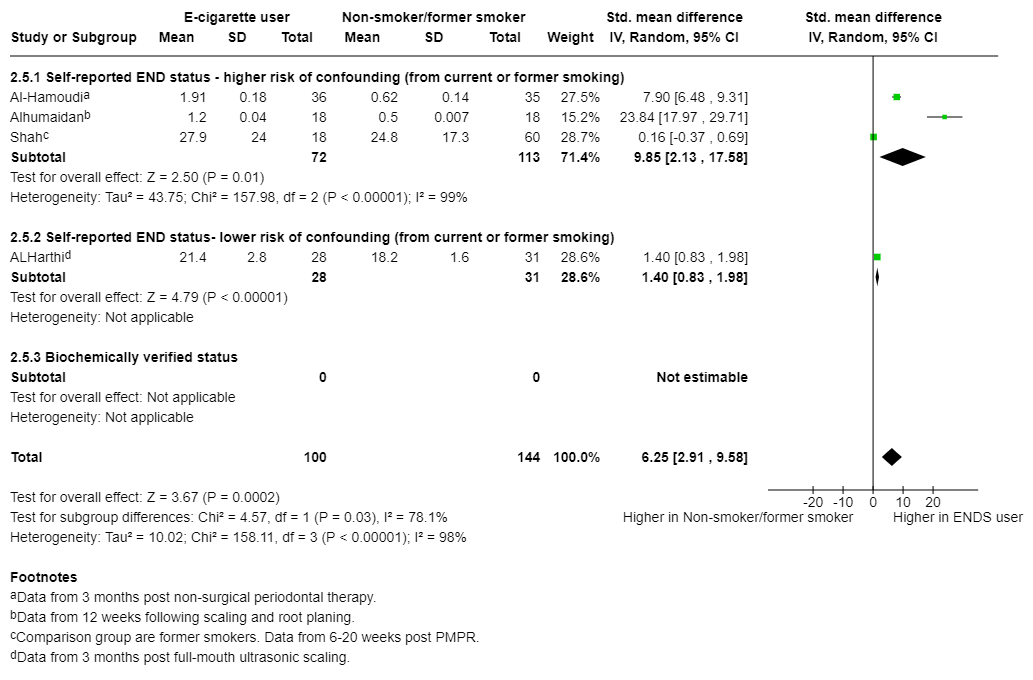


Figure S18. Forest plot of Plaque Index (PI) comparison for ENDS users verses smokers for post-intervention data: sub-grouped by risk of tobacco smoking confounding in ENDS group.


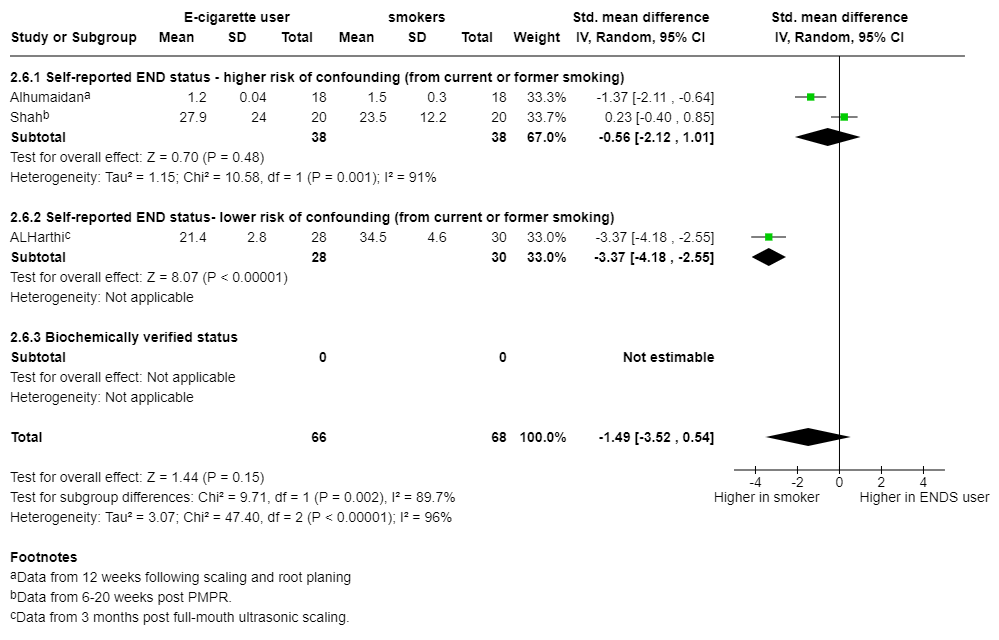


Figure S19. Funnel plot of BOP analysis: ENDS verses smokers (cross-sectional data) from Figure 3B.


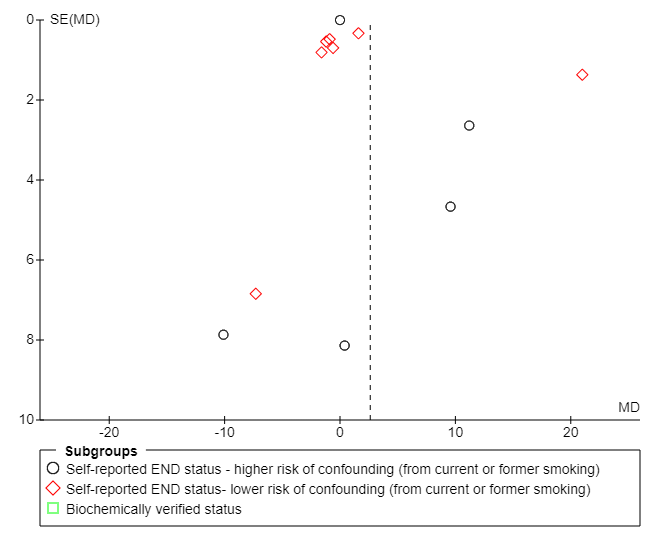


Figure S20. Funnel plot of PI analysis: ENDS verses non-smokers/former smokers (cross-sectional data) from Figure 4A.


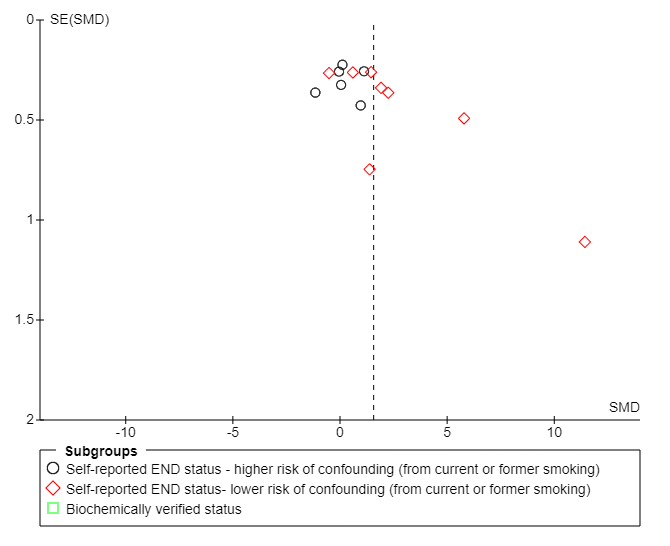


Figure S21. Funnel plot of PI analysis: ENDS verses smokers (cross-sectional data) from Figure 4B.


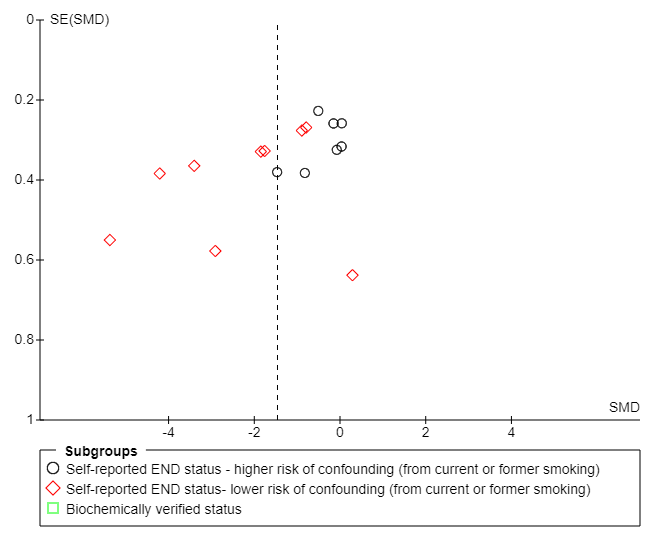


Figure S22. Funnel plot of CAL analysis: ENDS verses non-smokers/former smokers (cross-sectional data) from Figure S5.


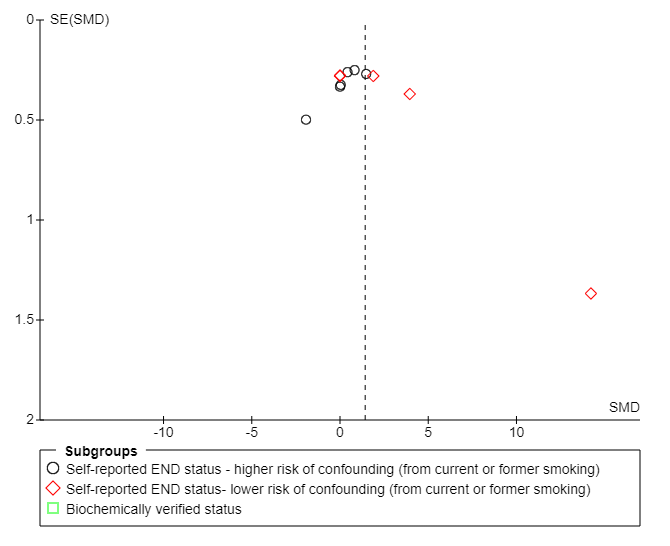


Figure S23. Funnel plot of CAL analysis: ENDS verses smokers (cross-sectional data) from Figure S6.


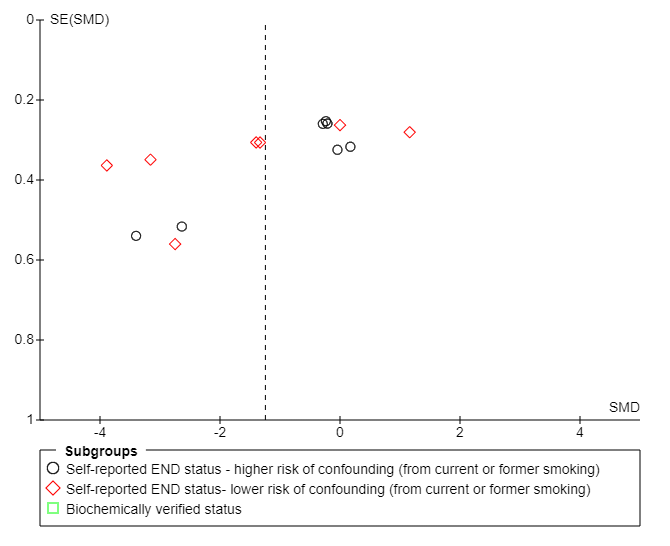


# 9. Figure S24. Geography of included studies.


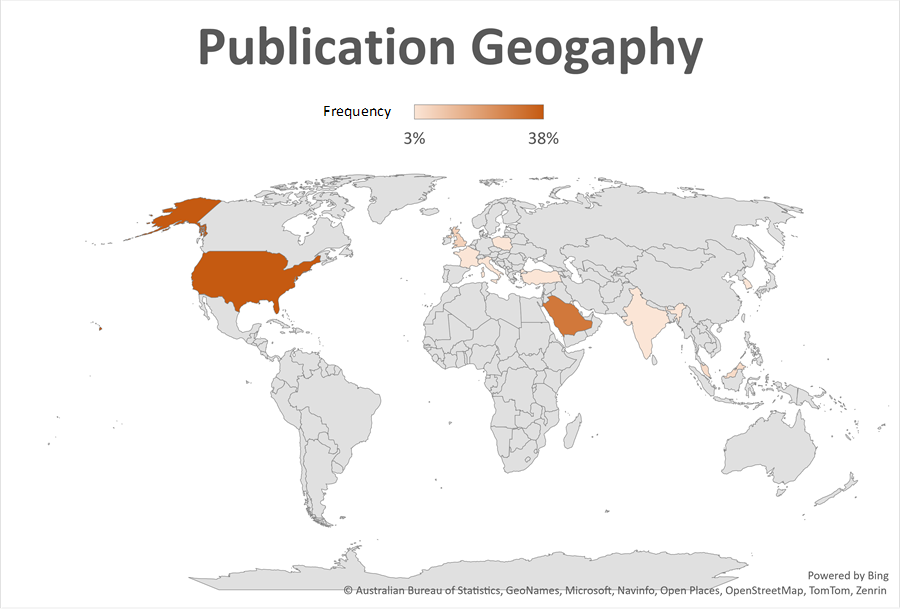


# 10. Adapted NOS RoB tools

## 10.1 NEWCASTLE - OTTAWA QUALITY SCALE ADAPTED FOR CASE CONTROL STUDIES FOR ENDS STUDIES ON PERIODONTAL HEALTH

**Selection**

1) Is the case definition adequate?

a) yes, with independent validation **🟑**

b) yes, eg record linkage or based on self reports

c) no description

2) Representativeness of the cases

a) consecutive or obviously representative series of cases **🟑**

b) potential for selection biases or not stated

3) Selection of Controls

a) community controls **🟑**

b) hospital controls

c) no description

4) Definition of Controls

a) no history of disease (endpoint) **🟑**

b) no description of source

**Comparability**

- 1. Comparability of subjects in different outcome groups on the basis of design or analysis. Confounding factors controlled.

a) Data/ results adjusted for relevant predictors/risk factors/confounders. In particular has tobacco smoking confounding been controlled for with bio-chemical verification to confirm no current tobacco smoking. Self-reported never smoker (i.e. not a former smoker) **🟑🟑**

b) Data/results not adjusted for all relevant confounders/risk factors/information not provided. Rely on self-reporting of non-smoking. Former smokers included in ENDS group

**Exposure**

1) Ascertainment of exposure

a) Objectively measured exposure **🟑🟑**

b) Self-reported exposure and/or salivary/GCF cotinine levels analysed **🟑**

c) No details provided

2) Same method of ascertainment for cases and controls

a) yes **🟑**

b) no

3) Non-Response rate

a) same rate for both groups **🟑**

b) non respondents described

c) rate different and no designation

**Case-control Studies Scores:**

Very Good Studies: 9-10 points

Good Studies: 7-8 points

Satisfactory Studies: 5-6 points

Unsatisfactory Studies: 0 to 4 points

## 10.2 NEWCASTLE - OTTAWA QUALITY SCALE ADAPTED FOR COHORT STUDIES FOR ENDS STUDIES ON PERIODONTAL HEALTH

**Selection**

1) Representativeness of the exposed cohort

1. Truly representative of the average in the target population. **🟑** (all subjects or random sampling)
2. Somewhat representative of the average in the target group. **🟑** (non-random sampling)
3. Selected group of users/convenience sample.
4. No description of the derivation of the included subjects

2) Selection of the non exposed cohort

a) drawn from the same community as the exposed cohort **🟑**

b) drawn from a different source

c) no description of the derivation of the non exposed cohort

3) Ascertainment of exposure

- 1. Objectively measured exposure **🟑🟑**
  2. Self-reported exposure and/or salivary/GCF cotinine levels analysed **🟑**
  3. No details provided

4) Demonstration that outcome of interest was not present at start of study

a) yes **🟑**

b) no

**Comparability**

- 1. Comparability of subjects in different outcome groups on the basis of design or analysis. Confounding factors controlled.

a) Data/ results adjusted for relevant predictors/risk factors/confounders. In particular has tobacco smoking confounding been controlled for with bio-chemical verification to confirm no current tobacco smoking. Self-reported never smoker (i.e. not a former smoker) **🟑🟑**

b) Data/results not adjusted for all relevant confounders/risk factors/information not provided. Rely on self-reporting of non-smoking. Former smokers included in ENDS group

**Outcome**

1) Assessment of outcome:

- 1. Blinded clinical assessment **🟑🟑**
  2. Independent objective validated laboratory methods **🟑🟑**
  3. Non-blinded clinical assessment. **🟑**
  4. Unblinded objective validated laboratory methods. **🟑🟑**
  5. Used non-standard or non-validated laboratory methods with gold standard. **🟑**
  6. Validated survey/questionnaire **🟑🟑**
  7. Unvalidated survey/questionnaire **🟑**
  8. No description/non-standard laboratory methods used.

2) Was follow-up long enough for outcomes to occur

a) yes (select an adequate follow up period for outcome of interest) **🟑**

b) no

3) Adequacy of follow up of cohorts

a) complete follow up - all subjects accounted for **🟑**

b) subjects lost to follow up unlikely to introduce bias - small number lost, or description provided of those lost) **🟑**

c) large number lost and no description of those lost

d) no statement

**Cohort Studies Scores:**

Very Good Studies: 10-11 points

Good Studies: 8-9 points

Satisfactory Studies: 6-7 points

Unsatisfactory Studies: 0 to 5 points

## 10.3 NEWCASTLE-OTTAWA SCALE ADAPTED FOR CROSS-SECTNAL FOR ENDS STUDIES ON PERIODONTAL HEALTH

**Selection:**

1) Representativeness of the exposed cohort

1. Truly representative of the average in the target population. **🟑** (all subjects or random sampling)
2. Somewhat representative of the average in the target group. **🟑** (non-random sampling)
3. Selected group of users/convenience sample.
4. No description of the derivation of the included subjects
   1. Sample size
5. Justified and satisfactory (including sample size calculation). **🟑**
6. Not justified.
7. No information provided
   1. Non-respondents
8. Proportion of target sample recruited attains pre-specified target or basic summary of non-respondent characteristics in sampling frame recorded. **🟑**
9. Unsatisfactory recruitment rate, no summary data on non-respondents.
10. No information provided
    1. Ascertainment of the exposure (risk factor)
11. Objectively measured exposure**🟑🟑**
12. Self-reported exposure and/or salivary/GCF cotinine levels analysed **🟑**
13. No details provided

.

**Comparability**

- 1. Comparability of subjects in different outcome groups on the basis of design or analysis. Confounding factors controlled.

a) Data/ results adjusted for relevant predictors/risk factors/confounders. In particular has tobacco smoking confounding been controlled for with bio-chemical verification to confirm no current tobacco smoking. Self-reported never smoker (i.e. not a former smoker) **🟑🟑**

b) Data/results not adjusted for all relevant confounders/risk factors/information not provided. Rely on self-reporting of non-smoking. Former smokers included in ENDS group

**Outcome:**

1) Assessment of outcome:

1. Blinded clinical assessment **🟑🟑**
2. Independent objective validated laboratory methods **🟑🟑**
3. Non-blinded clinical assessment. **🟑**
4. Unblinded objective validated laboratory methods. **🟑🟑**
5. Used non-standard or non-validated laboratory methods with gold standard. **🟑**
6. Validated survey/questionnaire **🟑🟑**
7. Unvalidated survey/questionnaire **🟑**
8. No description/non-standard laboratory methods used.
   1. Statistical test:
9. Statistical test used to analyse the data clearly described, appropriate and measures of association presented including confidence intervals and probability level (p value). **🟑**
10. Statistical test not appropriate, not described or incomplete.

**Cross-sectional Studies Scores:**

Very Good Studies: 9-10 points

Good Studies: 7-8 points

Satisfactory Studies: 5-6 points

Unsatisfactory Studies: 0 to 4 points

These tools have been adapted from the Newcastle-Ottawa Quality Assessment Scale for cohort and case-control studies to provide quality assessment of cross sectional studies and studies investigating the effects of ENDS use on periodontal health^^[[1]](#footnote-2)^^.

# 11. Table S4. Summary of previous systematic reviews on ENDS use and periodontal health

| Title | Authors | Year | Journal | No of studies included | RoB Analysis & Quality Assessment (QA) | Results analysis | Meta-analysis: Studies pooled or stratified based on confounding risk | Conclusion |
| --- | --- | --- | --- | --- | --- | --- | --- | --- |
| The impact of electronic and conventional cigarettes on periodontal  health—a systematic review and meta‑analysis | Thiem et al. | 2023 | Clin Oral Investig. | 16 | Cohort and case-control NOS used for cohort, case-control, and cross-sectional studies  Select studies assessed using GRADE | Meta-regressions were employed to examine the impact of various moderators on the studies included in the meta-analyses | Pooled | E-cigarette use might be a healthier alternative to cigarette smoking in terms of periodontal health, but it still has harmful effects on the periodontal tissues |
| Evaluation of periodontal indices among non-smokers, tobacco, and e-cigarette smokers: a systematic review and network meta-analysis | Pesce et al. | 2022 | Clin Oral Investig. | 5 | NIH quality assessment tool for observational cohort and cross-sectional studies | Assumptions for network meta-analysis, including similarity, transitivity, and consistency, were checked by qualitatively assessing study characteristics and statistically investigating consistency between direct and indirect comparisons | Pooled | Periodontal parameters were similar between non-smokers and e-cigarette users, with traditional smokers showing the worst indices. Both e-cigarette users and traditional smokers exhibited reduced BoP |
| Effects of Vape Use on Oral Health: A Review of the Literature | Iacob et al. | 2024 | Medicina (Kaunas) | 8 | No RoB analysis or QA assessment | No quantitative analysis and results presented as a narrative | N/A | Vaping is linked to a higher risk of periodontitis and caries, with users experiencing more oral problems than non-smokers, though less severe than those of traditional smokers. |
| The impact of vaping on periodontitis: A systematic review | Figueredo et al | 2021 | Clin Exp Dent Res. | 8 | JBI critical appraisal checklist tool | A linear mixed model was used to estimate the fixed effects of vaping on clinical measurements, with mean values weighted by their standard deviations to assess the impact of vaping as a factor | N/A | While evidence is limited, available results suggest vaping may contribute to increased periodontal destruction and the development of periodontitis |
| Periodontal and Peri-Implant Health Status in Traditional vs. Heat-Not-Burn Tobacco and Electronic Cigarettes Smokers: A Systematic Review | D’Ambrosio et al. | 2022 | Dent J (Basel) | 18 | ROBINS-I | The heterogeneity of the included studies and the absence of randomized controlled trials prevented a meta-analysis therefore, results are presented as a narrative | N/A | E-cigarettes may cause fewer inflammatory signs of periodontitis than traditional tobacco, but both e-cigarettes and Heat-Not-Burn products, which contain nicotine, can still negatively affect periodontal and peri-implant health |
| Effects of e-cigarette smoking on periodontal health A scoping review | Charde et al. | 2024 | PLOS Glob Public Health | 23 | No RoB analysis or QA assessment | No quantitative analysis and results presented as a narrative | N/A | Vaping may exacerbate periodontal disease by altering the host response and increasing inflammatory cytokines, with clinical studies showing worse periodontal health and treatment outcomes in e-cigarette users compared to non-smokers, though less severe than smoking. |

1. Herzog R, et al. Is Healthcare Workers’ Intention to Vaccinate Related to their Knowledge, Beliefs and Attitudes? A Systematic Review. *BMC Public Health* 2013 **13**:154 [↑](#footnote-ref-2)
